# Validation and psychometric properties of the Somatic and Psychological HEalth REport (SPHERE) in a young Australian-based population sample using non-parametric item response theory

**DOI:** 10.1186/s12888-017-1420-1

**Published:** 2017-08-01

**Authors:** Baptiste Couvy-Duchesne, Tracey A. Davenport, Nicholas G. Martin, Margaret J. Wright, Ian B. Hickie

**Affiliations:** 1 Queensland Brain Institute, the University of Queensland, Brisbane, Australia; 2 Centre for Advanced Imaging, the University of Queensland, Brisbane, Australia; 3 Genetic Epidemiology, QIMR Berghofer Medical Research Institute, Brisbane, Australia; 4 Brain and Mind Centre, The University of Sydney, Sydney, Australia

**Keywords:** Depression, Anxiety, Chronic fatigue, Non-parametric item response theory, Test-retest, Psychometrics, DSM-IV, Heritability, Twins

## Abstract

**Background:**

The Somatic and Psychological HEalth REport (SPHERE) is a 34-item self-report questionnaire that assesses symptoms of mental distress and persistent fatigue. As it was developed as a screening instrument for use mainly in primary care-based clinical settings, its validity and psychometric properties have not been studied extensively in population-based samples.

**Methods:**

We used non-parametric Item Response Theory to assess scale validity and item properties of the SPHERE-34 scales, collected through four waves of the Brisbane Longitudinal Twin Study (*N* = 1707, mean age = 12, 51% females; *N* = 1273, mean age = 14, 50% females; *N* = 1513, mean age = 16, 54% females, *N* = 1263, mean age = 18, 56% females). We estimated the heritability of the new scores, their genetic correlation, and their predictive ability in a sub-sample (*N* = 1993) who completed the Composite International Diagnostic Interview.

**Results:**

After excluding items most responsible for noise, sex or wave bias, the SPHERE-34 questionnaire was reduced to 21 items (SPHERE-21), comprising a 14-item scale for anxiety-depression and a 10-item scale for chronic fatigue (3 items overlapping). These new scores showed high internal consistency (alpha > 0.78), moderate three months reliability (ICC = 0.47–0.58) and item scalability (Hi > 0.23), and were positively correlated (phenotypic correlations *r* = 0.57–0.70; rG = 0.77–1.00). Heritability estimates ranged from 0.27 to 0.51. In addition, both scores were associated with later DSM-IV diagnoses of MDD, social anxiety and alcohol dependence (OR in 1.23–1.47). Finally, a post-hoc comparison showed that several psychometric properties of the SPHERE-21 were similar to those of the Beck Depression Inventory.

**Conclusions:**

The scales of SPHERE-21 measure valid and comparable constructs across sex and age groups (from 9 to 28 years). SPHERE-21 scores are heritable, genetically correlated and show good predictive ability of mental health in an Australian-based population sample of young people.

**Electronic supplementary material:**

The online version of this article (doi:10.1186/s12888-017-1420-1) contains supplementary material, which is available to authorized users.

## Background

**Fig. 1:**
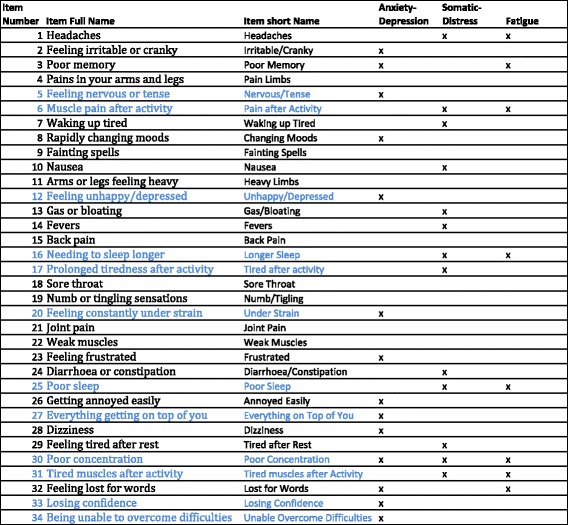
Items and scales of the SPHERE-34. Items’ short names are used through this manuscript. Some items may be included in several scales as indicated by multiple “x” in some rows. Items from the shorter SPHERE-12 appear in *blue*. Each scale of the SPHERE-12 comprises six items, which were created to provide a screening tool for common psychological and somatic distress in general practice [1, 85–87]. The two dimensional picture of the Australian population for the SPHERE-12 showed good psychometric properties and very high sensitivity for current and life-time major depression, anxiety and neurasthenia as assessed by DSM-III and DSM-IV [1, 86]. In addition, it was a good predictor of disability (as measured using the Brief Disability Questionnaire [88]), psychiatric morbidity [89] and doctor’s rating of psychological risk [1], which has led to its use in research and medical practice in Australia [97]

The Somatic and Psychological HEalth REport (SPHERE) provides an assessment of common symptoms of mental distress and persistent fatigue by self-report [1]. The 34 items of the SPHERE (SPHERE-34) were selected from four widely used clinical assessments of mental health, based on their predictive ability [1]. Anxiety and depression items were selected from the General Health Questionnaire [2], chronic fatigue from the Schedule of Fatigue and Anergia [3], neurasthenia from the Illness, Fatigue and Irritability Questionnaire [4], and somatisation items from the Diagnostic Interview Schedule (DSM)-III-R. Participants respond to each of the 34 items, choosing from one of three fixed options (“sometimes/never”, “often”, “most of the time” coded 0, 1 and 2 when calculating sum score) to describe the frequency of their symptoms over the “past few weeks”. While three subscales can be extracted: anxiety-depression, somatic distress and persistent fatigue (Fig. 1), these are assumed to represent overlapping constructs that underpin common mental disorders. Neurasthenia also used to be measured from the SPHERE-34 questionnaire but we do not consider it here, due to the progressive abandonment of the concept in psychiatry [5].

Except for an earlier paper from our group, where we showed that the anxiety-depression and somatic-distress subscales of the SPHERE-34 are moderately heritable (~40%) and correlated (phenotypic correlation of 0.42, genetic correlation of 0.87) [6], there has been no detailed assessment of the psychometric properties of this questionnaire outside clinical settings. This is important, as these properties may not generalise to population samples [7]. Here, we used Item Response Theory (IRT) to assess the validity and the psychometric properties of SPHERE-34 data collected in a large Australian-based population sample of young people [8, 9].

IRT origins trace back to the 1940s [10–12] and is a very popular framework for the validation of questionnaires, given the simplicity of model formulation and the numerous theoretical developments (chronologically: normal ogive, Rasch model, two and three-parameter logistic models, extensions for polytomous items, non-parametric IRT) [13–15]. There are two main advantages of IRT over classical test theory – it explicitly models the items’ properties and uses them to perform maximum likelihood (ML) estimation of the latent trait based on the individuals’ responses to the questionnaire. This provides an IRT score that takes into account the difficulty and discrimination of each item, often resulting in a more accurate estimation of ability, compared to using a sum of the items (sum score) (see [16, 17] or [18] for examples and simulations). That said, the sum score is also a (consistent asymptotically normal) estimate of the latent trait [19], and its use may be preferred to communicate test performances, and for use outside of a research context for obvious reasons of simplicity in calculation and interpretation [20].

In IRT, a scale of items (or questions) requires three hypotheses to be met: Unidimensionality: there is a single latent dimension (or trait) θ underlying a set of items; Conditional Independency: items are conditionally independent given the latent trait θ; Monotonicity of the Item Response Step Function: the probability of having a symptom, knowing the latent trait θ, is a growing function in θ. Conceptually, unidimensionality of a set of items is never verified, as several abilities are required to answer even the most simple question (e.g. reading ability, memory). Several tests have been proposed to assess unidimensionality [21], all testing H0 “Unidimensionality” vs. H1 “multidimensionality”. Thus, none of them can conclude that unidimensionality is verified; at best they conclude that it cannot not be invalidated. Furthermore, when large samples are considered, one would expect such tests to always reject the null hypothesis of unidimensionality. Similar criticisms can be formulated about testing for conditional independence (see [22] about necessary but not sufficient conditions for conditional independence). Consequently, we excluded items that did not satisfy the hypothesis of monotonicity or might be the most influenced by secondary abilities (see Methods below). However, we assumed that unique psychological dimensions could explain most of the responses to each subscale. Finally, we also assumed conditional independence: the answer to one item is not dependent on any other answer.

Nowadays, more than a dozen different IRT models for polytomous items have been proposed [23–25] that differ in hypotheses (definition of the IRSF) and properties of the final score [23–26]. Models can be classified into parametric (PIRT: IRSF are assumed to be logistic) and non-parametric IRT models (NIRT: no constraint on the shape of the IRSF). Here we chose to use NIRT models for several reasons. Firstly, in the absence of prior information about item properties, NIRT models do not assume the IRSF to be logistic. Items with non-logistic IRSF have been reported for depression scales, with NIRT leading to improved model fit and fewer items excluded [27]. Secondly, they allow a better diagnosis of the item properties by detecting local violations of monotonicity or local variations in item discrimination and bias [28, 29]. Thirdly, they offer user-friendly and straightforward diagnostic tools by means of visual inspection [28], and are less computationally demanding than PIRT by combining kernel regression and fast Fourier transform [16, 29]. Lastly, despite being more general than PIRT models, NIRT models have similar properties of stochastic ordering by the sum score [25, 26].

For the present study we merged the somatic-distress and fatigue scales into a “chronic fatigue” subscale, as they appeared to be driven by the same genetic factors (rG > 0.97, phenotypic correlation above 0.9 using the SPHERE-34 definition, see Additional file 1). This choice is consistent with the definition of the short version of the questionnaire (12 items, SPHERE-12, created for screening in general practice [1]), composed of two scales: psychological distress and somatic distress. Measuring both fatigue and depression could prove of great interest in psychiatric research, where it is known that they are highly comorbid [30] and often indicate a greater functional impairment when they co-occur [31]. Twin research further showed that depression and fatigue were strongly genetically correlated [32, 33], with however genetic and environmental factors specific to chronic fatigue [32, 33]. Causal relationships between depression and fatigue remain equivocal [31] with two studies reporting non-causal genetic relationships [33, 34]. Further research requires validated questionnaires that measure both dimensions and are suitable for longitudinal studies in the general population. Here, we use IRT to develop such scales from the SPHERE-34 questionnaire and we further report the psychometric properties, heritability, 3 months test-retest and association of the scores with DSM-IV diagnoses.

More precisely, we started from the depression-anxiety and fatigue scales previously defined [1] and excluded items responsible for bias in the score distribution and participant ordering (i.e. non-monotonic), as well as poorly contributing items with low discrimination. We also tried to improve the scale(s) stability and precision by including unused items from the former neurasthenia scale that showed good discrimination. Next, we investigated whether the new SPHERE scores (SPHERE-21) measured similar constructs across both age and sex, to ensure that any later differences observed across groups represent true differences in liability. Then we investigated the impact of the new scales definition on the scores reliability (3-months test-retest), internal consistency [35] and scalability (Loevinger’s Coefficients [36]). In addition, we estimated the heritability of the new depression and fatigue scales for each age group together with their genetic, environmental and phenotypic correlations. Finally, in a reasonably large subsample we assessed the predictive ability of the new SPHERE scores by examining the association of age specific SPHERE-21 scores with mental health lifetime diagnoses collected in early adulthood. We concluded with a post hoc comparison of the SPHERE-21 and Beck Depression Inventory (BDI) properties.

## Methods

### SPHERE questionnaires in the Brisbane longitudinal twin study

**Fig. 2:**
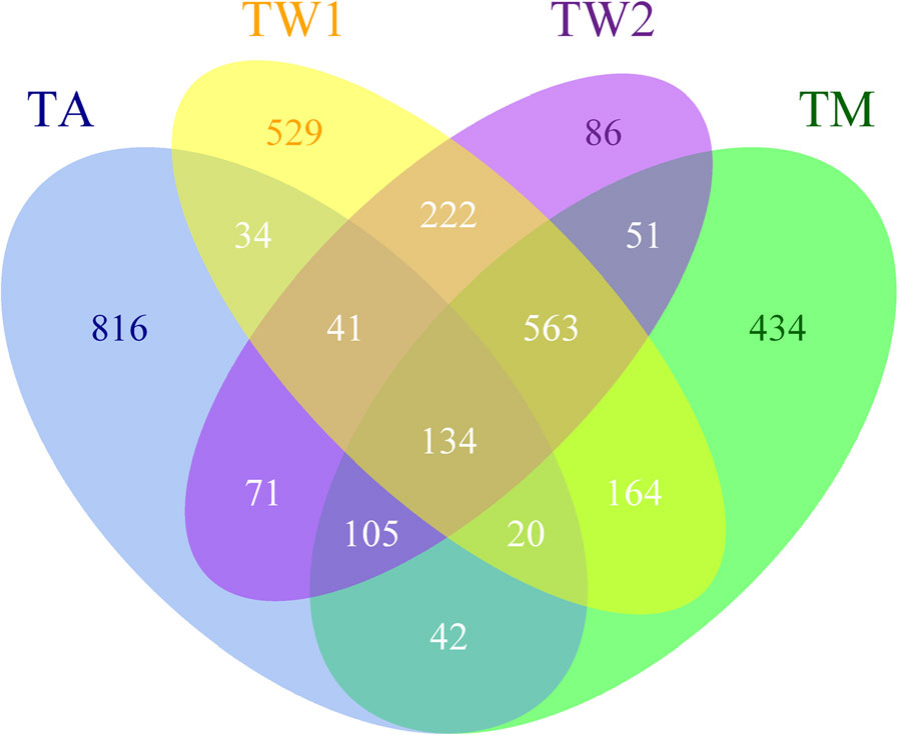
Venn diagram of the four waves of the BLTS that included the SPHERE-34

SPHERE-34 was administered as part of three main projects that make up the Brisbane Longitudinal Twin Study (BLTS; also known as the Brisbane Adolescent Twin Study (BATS)) [8]. The first two waves of data were collected in the clinic, following an assessment of Melanocyte Naevi (moles) around the twelfth (TWin Mole study visit 1: TW1) and fourteenth birthday of the twins (TWin Mole study visit 2: TW2) [6, 37–39] with a third wave of data, also collected in the clinic, as part of the twin cognition project (Twin Memory, attention and problem solving, TM), mostly at age 16 years [6, 40–43]. The final wave of SPHERE-34 data was collected as part of a mailout, which included assessments for laterality, personality and reading, as well as smell and taste tests; the study is known as the Twin Adolescent (TA) study. Participants who were administered the SPHERE-34, as part of the TA study, were on average 18 years old [6, 44, 45]. In total, 3312 twins or siblings (individuals) were included in at least one of the four waves in which the SPHERE-34 was administered, and at each wave responses were available for >1200 individuals (TW1: 1707; TW2: 1273; TM: 1513 and TA: 1263). Almost half of the participants (44%) answered the questionnaire more than once (19% three times), with 134 individuals (4%) being assessed at all four waves (Fig. 2). Missingness was overall limited (maximal percentage missingness per item ranged from 0% to 0.6%, number of participants with missing items ranged from 0% to 4.0%; Additional file 2) and at each wave can be assumed to be at random, with exclusions having little impact on results and power (Additional file 2).

### Selection of unrelated individuals for IRT analysis

As the sample included twin pairs and siblings, and IRT still lacks methods able to model relatedness in samples, we selected unrelated individuals for the IRT analysis. Despite a significant reduction of the sample size, the familial pruning ensures unbiased confidence intervals of IRSF and facilitates the comparison across sexes and studies, in which the relatedness might confound the results. In order to maximise the number of observations included, we randomly selected one individual per family in each of the waves. To ensure sampling homogeneity of our pruned sample with the full sample, we iterated the random selection process 1000 times, keeping the sample with the most similar age mean, variance and sex frequency as the full sample.

For the across wave comparison (study Differential Item Functioning (DIF), see below), we chose to successively compare TM, TW1, and TW2 to TA, which we used as a benchmark. This approach maximised the number of observations used in NIRT model estimation, hence reducing confidence intervals. For each dataset, we only allowed unrelated individuals within and across waves. When multiple observations were available for a participant we preferentially selected the observation from the wave that had a smaller number of participants in order to obtain a comparable sample size across waves. We iterated the familial pruning and observation selection 100 times each, keeping the sample that included the most similar number of participants across the four waves.

In most of the resultant (pruned) samples, there were slightly more females (2–9%; Table 1). Mean age in TW1, TW2, TW and TA was 12, 14, 16 and 18 years respectively (Table 1). Age had a pseudo-normal distribution in TA but exhibited large peaks in the other three waves due to the smaller age dispersion. Pruned samples (to investigate study DIF) showed comparable age and sex distributions as the full samples (Table 1).

**Table 1:** Demographics of the full samples and sub-samples pruned for relatedness and/or longitudinal observations

Wave	*N*	Mean age	SD	Age range	% Females
TW1	1695	12.64	1.33	9–18	51%
TW1 pruned for relatedness	651	12.63	1.34	10–19	51%
TW2	1265	14.03	0.68	9–18	50%
TW2 pruned	602	14.02	0.68	10–18	50%
TM	1513	16.49	0.84	15–22	54%
TM pruned	683	16.48	0.83	16–22	53%
TA	1213	18.06	3.07	11–28	56%
TA pruned	592	18.27	3.13	12–26	56%
TA + TM pruned –one assessment wave/ individual	1117				55%
TA subset	543	18.67	3.02	12–26	56%
TM subset	574	16.42	0.78	16–22	54%
TA + TW2 pruned	1012				52%
TA subset	506	18.93	2.98	12–26	56%
TW2 subset	506	14.10	0.52	10–16	47%
TA + TW1 pruned	1149				53%
TA subset	585	18.39	3.12	12–26	55%
TW1 subset	564	12.50	1.15	10–19	50%

### Protocol of non-parametric IRT analysis

**Fig. 3:**
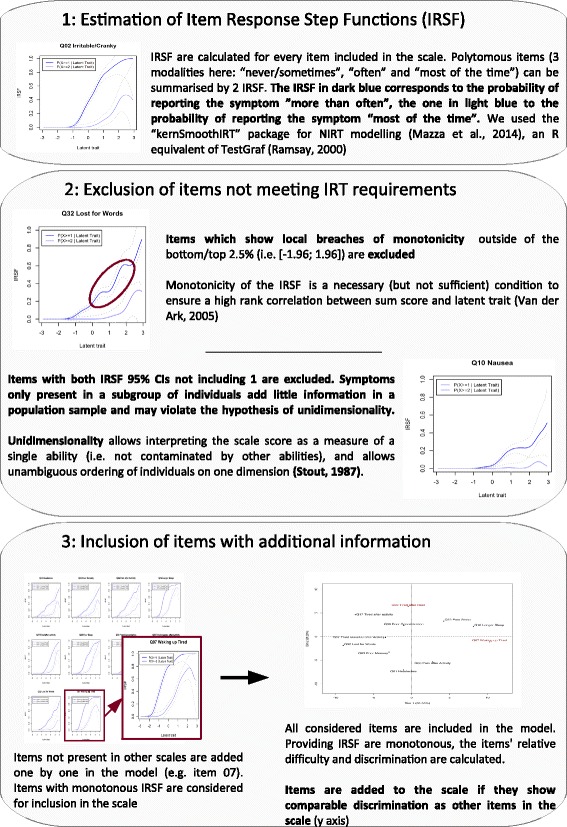
Protocol for SPHERE-34 scale development in the TA study

Redefining the SPHERE-34 subscales is an attempt to improve their properties by ensuring that the IRT hypothesis of monotonicity is met in practice but also by including, when possible, items frequently endorsed that inform on the individuals with low proficiency. We first examined its subscales in TA, the oldest cohort (mean age = 18 years, SD = 3.10), where we can assume questions were fully understood by most participants. Starting from subscales defined from clinical samples, we estimated the IRSF, excluding items not showing monotonic IRSF or specific to a subset of individuals, and included additional items (e.g. from the neurasthenia scale) that add information to the subscale (Fig. 3). An item’s relative difficulty and discrimination can be calculated using principal component analysis using the evaluation points of the expected item scores [16, 17]. The items are projected on the first two principal components. The first principal component often corresponds to the difficulty of the items, while the second principal component measures the items’ discrimination. Axes are detailed in each figure legend. Plots were created using the FactoMineR package [46, 47].

**Fig. 4:**
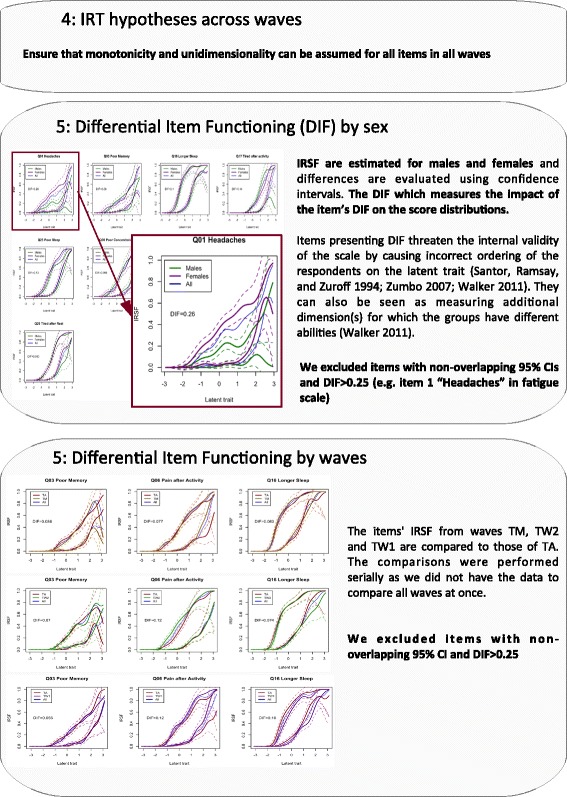
Protocol to study and limit DIF across sex groups and waves

Then, we studied sex DIF in all waves and excluded items responsible for large item bias (Fig. 4). Finally we evaluated the wave DIF to identify items behaving differently across studies or age groups (Fig. 4).

### Non-parametric IRT models and concepts

We used a non-parametric Graded Response Model [48, 49] that is the most general NIRT model for polytomic items [24, 26], while having the simplest and arguably the most plausible IRSF definition [50, 51]. Thus, hypothesis three of monotonicity becomes for each item j and each response *x* ∈ (0, 1, 2):

$$ P\left({X}_j\ge x\left|\theta \right.\right)\; is\;a\; monotonous\  nondecreasing\  function\  in\;\theta $$

With *X*_*j*_ the random variable of the score on item j. *P*(*X*_*j*_ ≥ 1|*θ*) and *P*(*X*_*j*_ ≥ 2|*θ*) are respectively the probabilities of reporting symptoms more than often (“often” or “most of the time”) or most of the time.

In NIRT, the absence of interpretable parameters (that define the logistic IRSF) forces one to rely on visual inspection of the IRSF or to rely on additional metrics [17, 52, 53] in order to describe or compare the functions. We used the “kernSmoothIRT” package [16] for NIRT modelling, which is an R equivalent of TestGraf [17]. It allows plotting the IRSF from which the hypothesis of monotonicity and the item properties could be visually appreciated. In addition, we calculated relative difficulty and discrimination of the items [17]. Using visual inspection and item bias summary statistics [17], we studied differential item functioning (DIF or item bias), present when individuals from different groups (e.g. sex, ethnicity, wave) with the same proficiency have different probabilities to endorse one item or one item category. DIF can cause an artificial score difference between groups and threatens the internal validity of the scale by causing incorrect ordering of the participants on the latent trait [53–55]. If DIF is strongly undesirable in the final score, it can also inform on the dimensionality of the scale. Indeed, items presenting DIF can be seen as measuring additional dimension(s) for which the groups have different abilities [55]. As a conclusion, study of DIF offers a partial check (limited to the groups considered) of the unidimensionality hypothesis in IRT. As DIF statistic we used the one implemented in Testgraf [17], which corresponds to the root mean square of the differences of IRSF over the latent trait, or more simply the mean absolute difference in probabilities of answering each item. We considered that DIF > 0.25 suggests a substantive difference of abilities, with one group on average 25% more likely to report one symptom. Thus, we chose to exclude such items, provided the difference between IRSF was significant as indicated by the 95% confidence intervals (see Fig. 4).

In addition, some NIRT models have fewer measurement properties than some of their parametric counterparts [24–26]. A central property that allows inferences to be made on the latent trait is stochastic ordering of the latent trait (SOL) by the sum score. It states that the order of participants, as given by the item sum score, gives a stochastically correct ordering on the latent variable [25]. In theory, this property is only verified [24, 25] for very simple polytomous parametric models [56, 57] that force the slope of the IRSF to be equal across items and categories. However, there is practical evidence that SOL by the sum score is often verified [26, 51] when IRT hypotheses are met, enough items are present (>5) with a limited number of categories (<5) and similarly shaped IRSFs [26]. Thus, our use of NIRT models maximises goodness of fit to the data while allowing us to make inferences on the individual’s proficiency based on their questionnaire score.

Finally, we preferred the kernel estimation of IRSF, or “TestGraf approach” [16, 29], over Mokken Scale Analysis (MSA) [15, 36, 58], another NIRT approach that relies on Loevinger’s Coefficient [11, 36] to assess properties of items. Despite a simpler framework, through the use of predefined criteria and rules (see Methods below), MSA often results in more item exclusions and suffers from the lack of interpretability of Loevinger’s Coefficient that are reduced by low correlation to latent trait, redundant items, intersecting IRSF, low discrimination or non-monotonicity [15]. We report Loevinger’s Coefficients of the final scales in the psychometric section as a measure of scalability.

### New scores description, three months test-retest, internal consistency and Mokken scale analysis

Using the final scales definitions, we estimated the ML estimate of the latent trait, which is an efficient estimate of the individuals’ proficiency [17] and calculated the sum score (items scored 0, 1 or 2 for “sometimes/never”, “often”, “most of the time”) as a benchmark of score performance. We report the mean IRT and sum scores by sex for the four waves. To accommodate related individuals in the sample, the sex difference was tested using the “hglm” R package (fixed effect, Student’s t-test) [59]. A matrix of genetic relatedness was used to model the variance covariance structure of the sample. Such matrix was created using the “kinship2” package [60].

For test-retest evaluation, we included all unrelated participants with a test-retest period shorter than four months. This resulted in 52 participants with a median test-retest interval of 1.9 months (range 1 day-3.8 months), 27 (50%) of the participants were females. Median age was 14 years (range 12–18). Test-retest was calculated using intra-class correlations (ICC) from the R package “irr” (two-way consistency ICC) [61].

Two widely used metrics in questionnaire validation include Cronbach’s alpha [35] and Loevinger’s Coefficient [11]. Cronbach’s alpha, often known as internal consistency, measures the proportion of the variance in the scale attributable to a common factor [62]. Despite being reported in almost every scale description, many parameters (e.g. number of items, items inter-correlation, dimensionality) have been shown to influence the coefficient [62], making its interpretation difficult [62, 63]. However, it is commonly considered that alpha >0.7 suggests an acceptable consistency while alpha >0.9 may indicate presence of redundant items [63]. The use of Loevinger’s Coefficient (H), or “scalability” coefficient, was popularised in Mokken Scale Analysis [15, 36, 58], a NIRT approach, which relies on a set of metrics to investigate the items or scale properties. Leovinger’s Coefficient can be calculated between two items (Hij), between an item i and a scale (Hi), of for a whole scale (H). Under the assumption of monotonicity of the IRSF, it has been shown that Hij > 0 for all (i,j), and Hi > 0 [64], however the reciprocal does not hold, thus Loevinger’s Coefficient cannot be used to confirm monotonicity of the IRSF. In addition, Loevinger’s Coefficient is sensitive to population variance, item difficulty, discrimination and presence of redundant items in the scale making their interpretation also difficult [65, 66]. However, it is commonly accepted that items satisfying Hij > 0 for all (i,j), i ≠ j, and 0.3 < Hi < 0.4 form a “weak Mokken scale”. When 0.4 < Hi < 0.5 the scale is defined as “medium”, and when 0.5 < Hi the items form a “strong Mokken scale” [36, 64]. Internal consistency was calculated in R using the “psy” package [67], Loevinger’s Coefficients were calculated using the package “Mokken” [58]. For all scores in all studies, we report Cronbach’s alpha, number of Hij < 0, min(Hi).

### Composition of the twin sample for heritability, genetic and environmental correlations

To facilitate interpretation of age specific heritability and correlations across ages, we binned the observations by age, creating four age bins (9 to <13 years, 13 to <15 years, 15 to <17 years and 17 to <28 years), which were centred around the mean age for each wave. For those individuals where two SPHERE-34 assessments occurred close together, which resulted in two assessments for an individual in an age bin, we randomly selected one SPHERE-34 assessment (Table 2). Next, we restricted the family size to a maximum of three siblings (one twin pair and one sibling or non-identical trio), which led to the exclusion of 161 participants (additional siblings or identical trio). Thus the final sample for genetic analyses comprised 1382 individuals with a mean age of 12 years, 1371 individuals with a mean age of 14 years, 1508 with a mean age of 16 years and 887 with a mean age of 19 years. See Table 2 for number of complete trios, monozygotic (MZ) and dizygotic (DZ) twin pairs, twin-sibling pairs and singletons. The two younger age bins had an equivalent proportion of males and females (50%), while there were slightly more females in the two older age bins (15 to 16 years (54%); 17 to 28 years (58%)) (Table 3).

**Table 2:** Sample size and demographics for genetic analyses

Age Bins:			8 to 12 years	13 to 14 years	15 to 16 years	17 to 28 years
Total number of observations			1492	1552	1683	959
Number of repeated observations excluded (same participant with two questionnaires in age bin)			53	154	108	25
Number of observations from identical triplet or extra siblings (excluded)			57	27	67	47
Final sample size (individuals) for genetic analyses			1382	1371	1508	887
Incl.	N complete twin pairs		634	603	670	242
	Incl.	N MZ pairs	226	209	230	96
		N DZ pairs	408	394	440	146
		N extra sibling	84	114	89	62
	N twin-sibling pair		1	1	0	23
	N singletons		28	49	79	295
	Incl.	N twins	8	8	15	96
		N siblings	20	41	64	199
Mean age (SD) [range]			12.09 (0.41) [9–12]	14.15 (0.31) [13, 14]	16.16 (0.37) [15, 16]	19.69 (1.92) [17–25]
% Females			50%	50%	54%	58%

**Table 3:** Sample size, demographics and prevalence of individuals with SPHERE-34 and CIDI

		8 to 12 years	13 to 14 years	15 to 16 years	17 to 28 years
N stratified by age at SPHERE-34 assessment		709	907	1055	739
Demographics					
	Mean age at CIDI (SD)	21.9 (1.7)	22.9 (2.4)	23.2 (2.5)	28.9 (3.1)
	*N* (%) Females	415 (59%)	522 (58%)	641 (61%)	453 (61%)
Prevalence					
MDD	*N* (%)	118 (16.6%)	150 (16.5%)	172 (16.3%)	119 (16.1%)
	Mean age onset (SD)	17.6 (3.1)	18.2 (3.5)	18.9 (3.4)	22.7 (5.3)
Social anxiety	*N* (%)	133 (18.7%)	157 (17.3%)	182 (17.3%)	115 (15.5%)
	Mean age onset (SD)	12.2 (4.5)	12.4 (4.7)	11.9 (5.0)	12.2 (5.5)
Alcohol dependence	*N* (%)	169 (23.8%)	249 (27.4%)	300 (28.4%)	188 (25.4%)
Marijuana dependence	*N* (%)	37 (5.2%)	59 (6.5%)	55 (5.2%)	30 (4.1%)
Panic disorder	*N* (%) (with agoraphobia)	4 (0.6%)	7 (0.8%)	10 (0.9%)	3 (0.4%)
	Mean age onset (SD)	17.0 (2.7)	16.6 (2.9)	16.4 (3.8)	22.0 (3.6)
	*N* (%) (without agoraphobia)	13 (1.8%)	13 (1.4%)	12 (1.1%)	15 (2.1%)
	Mean age onset (SD)	14.9 (3.6)	15.1 (4.1)	16.6 (4.8)	20.8 (3.8)

### Heritability, genetic and environmental correlation between the scores

We used a twin and sibling design to partition the variance into additive genetic “A”, unique environment components “E” and either familial (common) environment “C” or dominant genetic “D” [68–71]. Heritability is defined as the proportion of trait variance explained by the additive genetic factor. The twin design relies on the fact that, for a heritable trait, the twin-pair correlation increases with the degree of genetic relatedness, resulting in higher twin correlations in the MZ group compared to the DZ group. Here, we included an additional sibling when available, which provides an increase in power for detecting A and C/D [72]. Finally, we included singletons (in studies TA and TM) that do not contribute to power for detecting A or C/D, but improve the stability of the estimates of means and variance. Analyses were performed in OpenMx 2.2.6 [71, 73] using full information maximum likelihood (FIML) to accommodate singletons and incomplete trios. We compared the fit of ACE vs. ADE models using the Akaike Information Criterion [74], and tested the significance of A, C/D and E fraction of variance using log-likelihood ratio test on nested models.

Prior variance component modelling, we tested the comparability of means, and variances across zygosity groups and siblings, to identify sampling issues and outliers that may bias the results [75]. In order to limit the number of tests and improve readability, we performed an omnibus test (likelihood ratio test, 20 degrees of freedom) that tests whether equating all means and variance results in a significant reduction of the model fit. In addition, we tested the effect of sex, age and study on the score means, and also whether the twin covariances suggested sex-specific heritability [76]. All significant covariates were included in subsequent analysis. For each age group, we reported the heritability of the scores (IRT and sum scores). Then, we fitted a bivariate model to estimate the genetic and environmental correlations between anxiety-depression and chronic fatigue.

### Collection of the DSM-IV clinical assessment

A later wave of the BLTS (“19up: the study mapping neurobiological changes across mental health stages”) [8] collected Composite International Diagnostic Interviews (CIDI) [77] that we used to compute DSM-IV diagnoses of major depressive disorder (MDD), social anxiety, alcohol and marijuana dependence (i.e. substance dependence), and panic disorder [8]. As of *June* 2016, a total of *2773* twins and siblings had completed the study, of *which 2041* had previously answered at least one SPHERE-34 questionnaire. *709* participants had a SPHERE-34 score collected between 8 and 12 years (mean age at CIDI = 21.9, SD = 1.7, 59% females), 907 with SPHERE-34 between 13 and 14 years (mean age at CIDI = 22.9, SD = 2.4, 58% females), 1055 with SPHERE-34 between 15 and 16 years (mean age at CIDI = 23.2, SD = 2.5, 61% females) and 739 who answered the questionnaire between 17 and 28 years (mean age at CIDI = 28.9, SD = 3.1, 61% females). Despite a later age at CIDI for individuals who completed the SPHERE-34 questionnaire after age 17 years, the prevalence of MDD, social anxiety and substance dependence were comparable to the other SPHERE-34 age bins (Table 3). This should prevent the association between SPHERE-34 and the DSM-IV diagnoses being confounded by censoring. Thus, different predictive abilities of age-specific SPHERE-34 scores can be attributed mostly to differences in age at questionnaire, rather than to age at CIDI. Age of onset is not available for substance dependence and only the age at initiation was collected.

### Association of new SPHERE scores with DSM-IV diagnoses

We estimated the increased risk of DSM-IV diagnoses (MDD, social anxiety, alcohol and marijuana dependence) associated with an increased SPHERE-21 score. We do not report results of association with panic disorder as low numbers made estimation of parameters impossible. Results are presented in the form of odds ratio, which are equivalent to relative risk estimates as disease prevalences in our sample match those of the general population. To accommodate related individuals in the sample, the model parameters were estimated using quasi-likelihood implemented in the “hglm” R package (fixed effects, Student’s t-tests) [59]. A matrix of genetic relatedness, created using the “kinship2” package [60], was used to model the variance-covariance structure of the sample. This approach provides unbiased estimates of the variance of the estimates, which prevents underestimating *p*-values. Sex, ages at SPHERE, age at CIDI and dummy variables for the SPHERE study waves were included as covariates in the model. Finally, we estimated the number of independent SPHERE-21 scores across all age bins (np) using the eigenvalues of the correlation matrix [78, 79]. We then used a Bonferonni significance threshold of 0.05/(np*4), four being the number of diagnoses tested, to avoid enforcing a too stringent significance threshold.

## Results and discussion

### Redefinition of the SPHERE scales in the sample of young adults (TA study)

#### Anxiety-depression scale

**Fig. 5:**
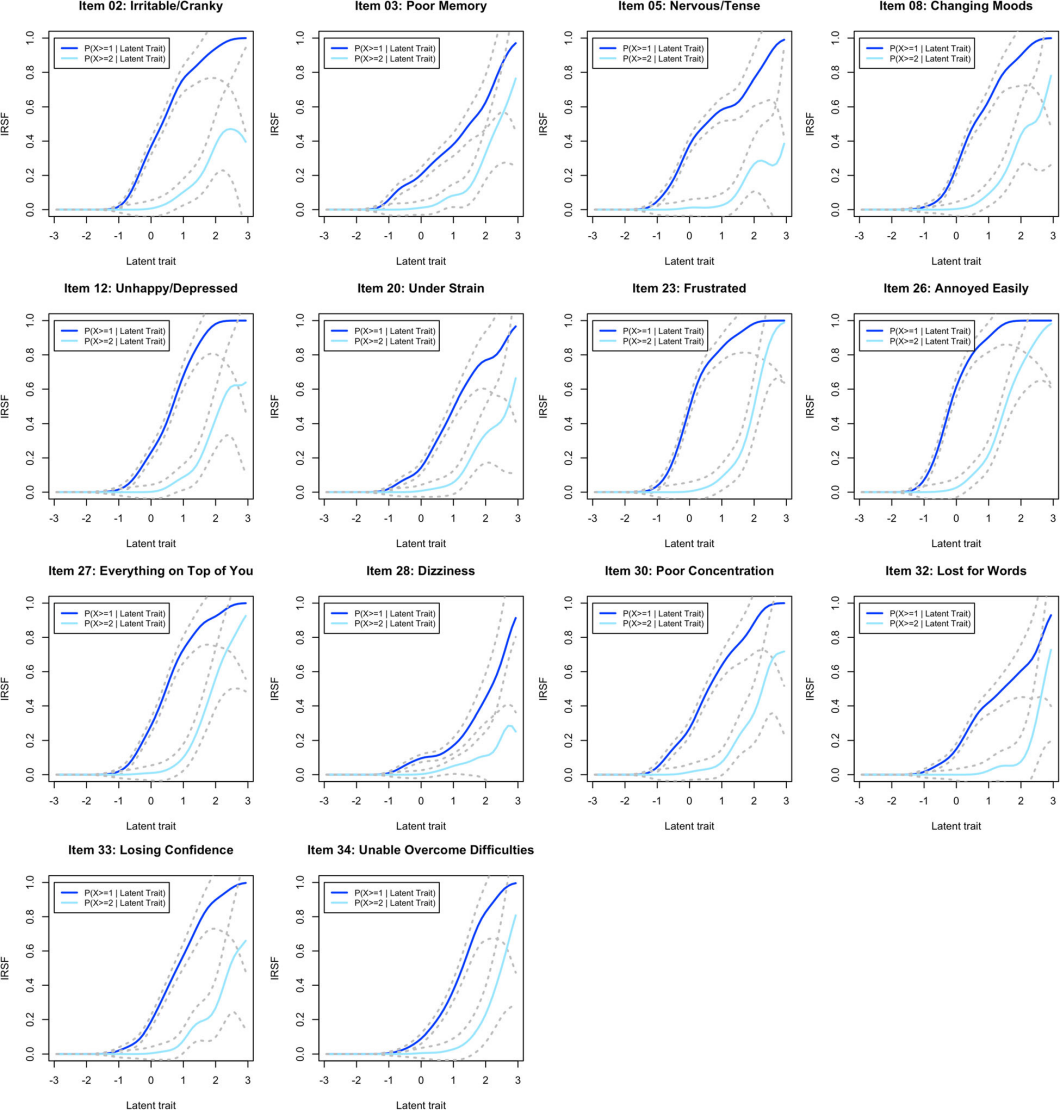
Response Step Functions of the 14 items proposed to measure anxiety-depression. For each item, two IRSF are calculated that correspond to the probability of having the symptom more than often (*dark blue line*) and the probability of having the symptom most of the time (*light blue line*). *Dotted lines* correspond to the 95% confidence interval of the IRSF. Using NIRT estimation, we do not constrain the IRSF to be logistic

**Fig. 6:**
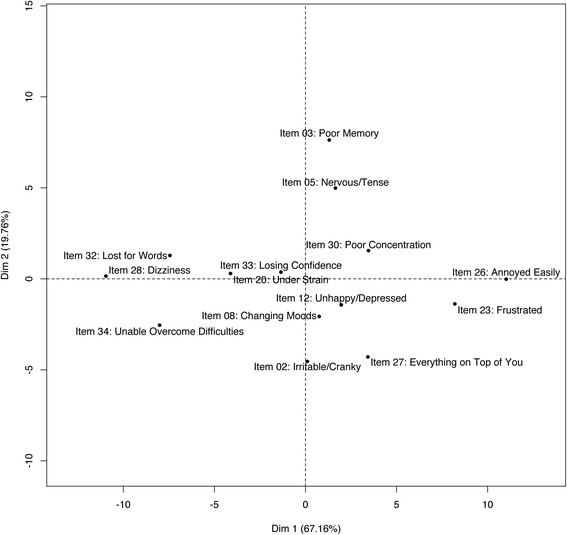
Difficulty and discrimination of the 20 items suitable for the anxiety-depression scale. The first principal component corresponds to the difficulty of the items, the easiest items being on the right (items 26 and 23). The second principal component measures the items’ discrimination (item 2 being the most discriminant)

All items of the original anxiety-depression scale showed monotonic IRSF in the normal range of the latent trait distribution. Item 5 (“Nervous/ tense”) presented the most obvious decrease of IRSF (Fig. 5), but this was limited to the top 2.5% of the distribution, which did not justify its exclusion. Additional items showed monotonous IRSF in the presence of the other 14 items and could be considered pertinent for the assessment of anxiety-depression: item 1 (“Headaches”), 7 (“Waking up tired”), 16 (“Longer sleep”), 17 (“Tired after activity”), 29 (“Tired after rest”), and item 31 (“Tired after activity”). However, these were all items from the somatic-distress or fatigue scales and we did not include them in the anxiety-depression scale to avoid artificially inflating the correlation between the 2 scores. Item 3 (“Poor memory”) was the least discriminant (Fig. 6) having the flattest IRSF, while item 2 (“Irritable/cranky”) was the most discriminant (steepest IRSF). Items 26 (“Annoyed easily”) and 23 (“Frustrated”) were the least difficult (Fig. 6) being endorsed by individuals with low proficiency (early elevation of IRSF, see Fig. 5), while item 28 (“Dizziness”) was the most difficult.

#### Chronic fatigue scale

**Fig. 7:**
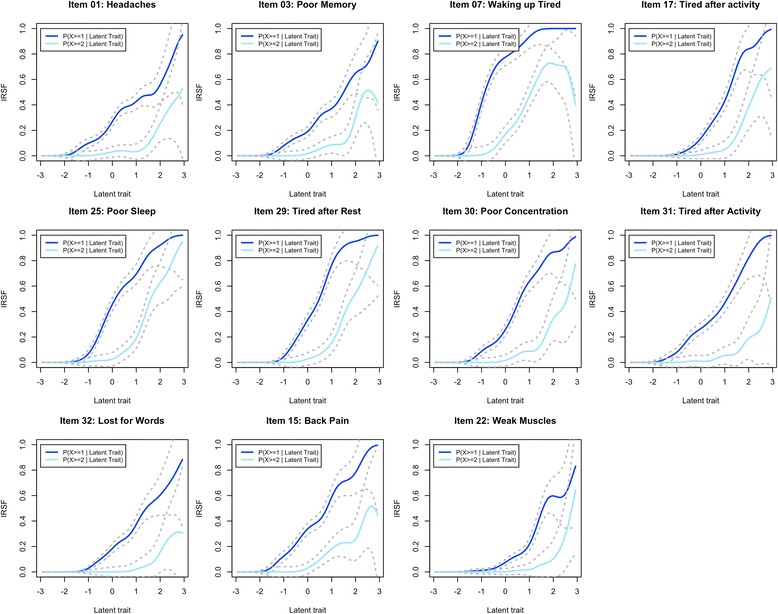
Response Step Functions of the 11 items suitable for the chronic fatigue scale

**Fig. 8:**
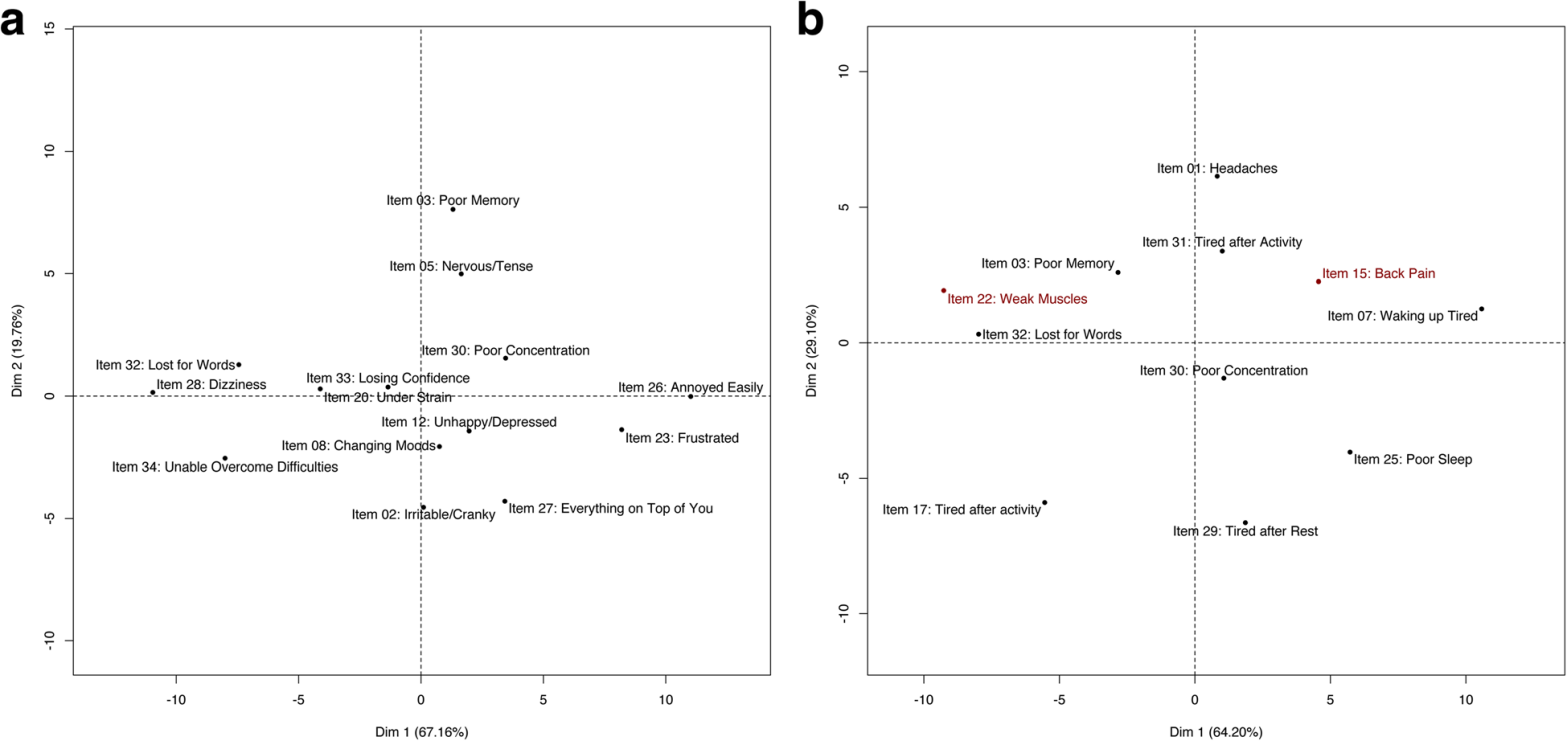
Difficulty and discrimination of the 11 items suitable for the somatic distress scale. The first principal component corresponds to the difficulty of the items, the easiest items being on the left (items 22 and 32). The second principal component measures the items’ discrimination (item 29 being the most discriminant, 1 the least discriminant). Added items appear in *red*

We started with the 15 items present in either the somatic-distress or fatigue scales (items 1, 3, 6, 7, 10, 13, 14, 16, 17, 24, 25, 29, 30, 31 and 32) and estimated the IRSF. Several items exhibited small local decreases in their IRSF (Additional file 3). However, this could be a consequence of the presence of items poorly correlated to the scale, leading to biased estimation of the latent trait. Indeed, items 14 (“Fevers”) and 24 (“Diarrhoea/ constipation”) were not often endorsed, even for individuals with very high latency (Additional file 3). For example, the estimated probability of reporting fevers “more than often” was below 0.4, and no participants reported fevers “most of the time” (Fig. 8). Thus, we excluded items 14 and 24 as they corresponded to symptoms rarely reported or only present in a subgroup, as suggested by 95% confidence intervals of the IRSF not reaching 1 (Additional file 3). We further excluded items 6, 10 and 16 for non-monotonicity and item 13 for its low endorsement. These exclusions resulted in smoother and monotonous IRSF for the nine remaining items (Additional file 4). Then, we considered relevant items not included in the anxiety-depression scale: items 15 (“Back pain”) and 22 (“Weak muscles”). After inclusion of these additional items, the IRSF remained monotonous (Fig. 7). Overall, item 1 (“Headaches”) was the least discriminant (Fig. 8) having the flattest IRSF, while items 17 (“Tired after activity”) and 29 (“Tired after rest”) exhibited the steepest IRSF (Figs. 7 and 8). Newly included items 15 and 22 were moderately discriminant, with item 22 being the most difficult in the scale, hence adding information on the individuals with extreme somatic-distress. These two items were included in the chronic fatigue scale.

### Differential item functioning across sex and study wave

#### Anxiety-depression scale

Sex DIF was moderate to low in all study waves and items, even if item bias might be slightly more pronounced in the TM study that shows a median DIF statistic of 0.13 (vs. 0.11 in TA, 0.065 in TW2 and 0.080 in TW1). Maximum sex DIF was found for item 33 (“Losing confidence”; DIF = 0.23) and item 27 (“Everything on top of you”; DIF = 0.22) in study TM. However, we kept these items in the scale as they did not show consistent DIF across studies, and their impact on the TM score would remain small (DIF < 0.25 and mostly overlapping 95% CIs, Additional file 5, Additional file 6, Additional file 7, and Additional file 8).

We observed a very limited DIF between waves (Additional file 9, Additional file 10, and Additional file 11) suggesting that the anxiety-depression scale measures the same latent construct across waves, hence age groups. Median item DIF was 0.087 for TM vs. TA, 0.10 for TW2 vs. TA and 0.088 in TW1 vs. TA. Items 12 (“Unhappy/ depressed”), 20 (“Under strain”) and 23 (“Frustrated”) consistently showed item bias above the median, TA participants being more likely to report the symptoms “more than often”, knowing the latent trait. However, these levels of DIF (0.13–0.24), which would have moderate impact on the scores, did not justify exclusion of these items.

#### Chronic fatigue scale

Monotonicity of the IRSF was observed for all items and waves, in the normal range of the chronic fatigue continuum (Additional file 12, Additional file 13, Additional file 14, and Additional file 15). The median sex DIF of the scale was 0.11 in the TA study, 0.12 in TM, 0.10 in TW2 and 0.092 in TW1, suggesting overall minor artificial sex differences in chronic fatigue scores (Additional file 12, Additional file 13, Additional file 14, and Additional file 15). However, we excluded item 1 (***“***Headaches***”***) which showed a high item bias (DIF = 0.26 in TA and 0.24 in TM, non-overlapping CIs: Fig. 8), with females more likely to report headaches when compared with males with the same latent score.

We observed very limited DIF between TM, TW2, TW1 and TA (Additional file 16, Additional file 17, and Additional file 18). Median DIF across items was 0.084 for TM vs. TA comparison, 0.090 for TW2 vs. TA and 0.069 in TW1 vs. TA. Item 15 (“Back pain”) was more frequently reported by participants of the TA study and showed the highest DIF in TW1 vs. TA and TW2 vs. TA (DIF = 0.21) but not in TM vs. TA (DIF = 0.084). However, the item did not meet the DIF exclusion criteria and we maintained it in the scale.

### Summary of NIRT analysis: The SPHERE-21 questionnaire

NIRT analysis showed that IRSF of the SPHERE-34 items were roughly logistic, varying in difficulty and discrimination (Figs. 6 and 8), sometimes exhibiting right asymptotes below 1 and local plateaus (Figs. 5, 7; Additional file 3 and Additional file 4). The latter would cause even the most complex PIRT model (four parameters logistic, with parameters measuring difficulty, discrimination, left and right asymptotes) to fit the data poorly. Using common PIRT model (e.g. two parameters logistic – modelling difficulty and discrimination only) would have resulted in poorer fit to the data, likely resulting in exclusion of more items. Overall, such exclusions would have led to smaller scales that tend to be less reliable and precise [66]. Finally, the IRSF left asymptotes were all 0, which suggests absence of guessing (i.e. no participants answering the questions at random). Thus we can infer that participants in all waves understood the questions (or answered by the negative when they did not understand).

The anxiety-depression scale was left unchanged after NIRT analysis. Across all four waves the anxiety-depression items met the IRT hypothesis of monotonicity. In addition, no item showed substantial DIF by sex or study wave suggesting the scale measures a consistent construct across groups and that sex or study wave differences observed arise mostly from true differences in latent trait.

On the other hand, we excluded six items from the chronic fatigue scale that were only endorsed by a fraction of the participants or did not satisfy the requirement of monotonicity of the IRSF. Two additional items, not present in the anxiety-depression scale, were added to improve the stability of the scale and/or the score distribution, as these provide information about individuals with low levels of chronic fatigue. Finally, we excluded item 1 (“Headaches”) that showed large sex DIF (in studies TA and TM), being more frequently reported by females, compared to males with the same proficiency. All other items of the chronic fatigue scale met DIF inclusion criteria. Overall low DIF suggests that the scales measure comparable constructs across sex and waves, hence age groups.

**Fig. 9:**
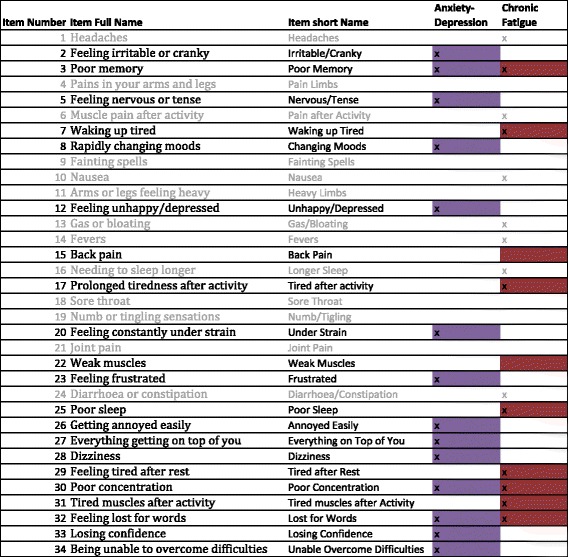
Summary of NIRT item validation and selection. Items from the original scales are indicated by an x. Items included in the new version of the scales are indicated by a rectangle of colour. Items not present in any scale after the reduction to 21 items appear in *light grey*

The final version of the SPHERE-21, which measures anxiety-depression (14 items) and chronic fatigue (10 items) is available in Additional file 19 (questionnaire) and Fig. 9 (scale definition). Three items are present in both scales (items 3 “Poor memory”, 30 “Poor concentration” and 32 “Feeling lost for words”).

We computed the IRT and sum scores of the two scales of SPHERE-21. As they both satisfy IRT hypotheses, contain enough items (>5), with a limited number of categories (<5) and similarly shaped Item Response Step Functions, stochastic ordering by the sum score can be assumed [26]. This allows inferring the ordering of the participants’ true abilities from the ordering of the SPHERE-21 sum score.

### SPHERE-21 mean scores, reliability, internal consistency and Mokken scale analysis

Using all observations available we tested for sex differences, after correcting for familial relatedness. Females had significantly higher anxiety-depression scores compared with males in study TM and TA (+0.4 and +0.5 for sum scores, *p*-values < 8.8E-4), but the difference was not significant at younger ages in TW2 and TW1 (after correction for multiple testing, Bonferroni correction). In addition, female’s reported lower chronic fatigue in study TW1 (−0.2 pt. in sum score *p*-value < 9.7E-5) but no significant differences survived multiple testing correction in the older waves (Table 4). Up to a quarter of the participants answered “never or sometimes” to all questions (22% in TA, 23% in TM, 25% in TW2 and 21% in TW1) yielding a sum score of 0 and an IRT score of −3. This proportion was lower for chronic fatigue (15% in TA, 14% in TM, 17% in TW2 and 19% in TW1).

**Table 4:** Mean SPHERE-21 IRT and sum score for each scale, wave and sex group

		Mean (SD)	Mean Females (SD)	Mean Males (SD)	Sex difference *p*-value
TA					
Anxiety-depression	IRT score	−0.39 (1.6)	−0.27 (1.6)	−0.56 (1.7)	**7.6E-4**
	Sum score	4.6 (4.8)	5.0 (4.9)	4.2 (4.6)	**8.8E-4**
Chronic fatigue	IRT score	−0.24 (1.5)	−0.19 (1.4)	−0.30 (1.5)	0.087
	Sum score	3.9 (3.4)	3.9 (3.4)	3.8 (3.5)	0.37
TM					
Anxiety-depression	IRT score	−0.44 (1.7)	−0.25 (1.7)	−0.67 (1.6)	**2.5E-6**
	Sum score	4.1 (4.5)	4.7 (5.0)	3.3 (3.7)	**9.3E-10**
Chronic fatigue	IRT score	−0.22 (1.5)	−0.15 (1.5)	−0.31 (1.4)	0.042
	Sum score	4.0 (3.4)	4.2 (3.6)	3.7 (3.2)	0.018
TW2					
Anxiety-depression	IRT score	−0.47 (1.7)	−0.47 (1.7)	−0.46 (1.7)	0.88
	Sum score	3.7 (4.3)	3.7 (4.2)	3.7 (4.4)	0.96
Chronic fatigue	IRT score	−0.28 (1.5)	−0.38 (1.5)	−0.18 (1.5)	0.031
	Sum score	3.6 (3.3)	3.4 (3.3)	3.8 (3.3)	0.079
TW1					
Anxiety-depression	IRT score	−0.4 (1.6)	−0.48 (1.7)	−0.30 (1.6)	0.026
	Sum score	4.1 (4.4)	3.9 (4.3)	4.4 (4.5)	0.023
Chronic fatigue	IRT score	−0.33 (1.6)	−0.48 (1.6)	−0.18 (1.5)	**2.5E-6**
	Sum score	3.5 (3.3)	3.3 (3.3)	3.8 (3.4)	**9.7E-5**

Here, we performed eight tests yielding a (conservative) Bonferonni-corrected significance threshold of 0.0063. Significant *p*-values after multiple testing correction appear in bold. All participants were used to produce this table. Relatedness was accounted for using mixed models when testing sex-differences

The IRT SPHERE-21 scores are moderately reliable: ICC = 0.47 [0.23, 0.66] for anxiety-depression and ICC = 0.57 [0.35, 0.73] for chronic fatigue. Reliability of chronic fatigue aligns with those of somatic-distress (ICC = 0.57 [0.37, 0.73]) or fatigue (ICC = 0.62 [0.42, 0.76]). Reliabilities of the IRT scores were higher (albeit non-significantly) than those of the sum scores (0.25 [−0.025, 0.49] for anxiety-depression, 0.49 [0.26, 0.67] for chronic fatigue). In addition, the internal consistency, as measured by Cronbach’s alpha, was greater than 0.7 for all scores (Table 5). Anxiety-depression has the highest internal consistency (alpha in 0.86–0.88), versus 0.78–0.79 for chronic fatigue.

**Table 5:** Cronbach’s alpha and Loevinger’s Coefficients of the SPHERE-21 anxiety-depression and chronic fatigue scales

		TW1	TW2	TM	TA
Anxiety-depression	alpha	0.88	0.87	0.87	0.86
	# Hij < 0	0	0	0	0
	Min(Hi)	0.29	0.24	0.28	0.24
	Items Hi < 0.3	3	3, 28	28	3
Chronic fatigue	alpha	0.79	0.79	0.78	0.79
	# Hij < 0	0	0	0	0
	Min(Hi)	0.24	0.26	0.25	0.23
	Items with Hi < 0.3	3, 15	3, 15, 31	3, 15	3, 15, 32

Alpha corresponds to Cronbach’s alpha; #Hij < 0 to the number of pairwise Loevinger’s Coefficient below 0 for (i,j) items; Min(Hi) is the minimal Loevinger’s Coefficient between an item i and the scale; Items Hi < 0.3 lists the item number corresponding to Hi < 0.3

Similarly, the pairwise Loevinger’s Coefficients (Hij) were all positive, indicating positive item correlation in each scale (Table 5). In addition, the Hi were also positive, which is expected when the hypothesis of monotonicity of the IRSF is met [64]. However, we note that the minimal Hi were all below 0.3 (items with Hi < 0.3, Table 5) and that items with low discrimination (e.g. items 3, 15, 31 in chronic fatigue, item 3 in anxiety-depression, see Figs. 6 and 8) would be excluded in Mokken Scale Analysis (MSA) [15]. Items 32 in chronic fatigue and 28 in anxiety-depression would also be excluded in MSA despite their good discrimination. Thus, one may prefer to use MSA for its simplicity, or when trying to reduce the length of a questionnaire. The counterpart being that MSA relies on rather arbitrary criteria (see [65] for further discussion on the interpretation of Loevinger’s coefficients) and, like PIRT, may reduce reliability and precision of the instrument [66].

### Heritability, genetic and environmental correlations between the SPHERE-21 scores

Covariate effect, twin-pair correlations and homogeneity of sampling across twin zygosity groups and siblings were investigated for IRT and sum scores in each age bin. Detailed results are available in Additional file 20. In summary, sex was nominally significant (*p*-value < 0.05) for most bins and scores, except for the anxiety-depression scores of the 13 and 14-year age group (*p*-values = 0.78 and 0.91). Females had lower anxiety-depression (−0.85 sum score, *p*-value = 4.8E-4) and chronic fatigue scores (−0.79 sum score, *p*-value = 4.5E-6) at age 8 to 12 years. At older ages, females had higher anxiety-depression (+1.52 sum score at 15 to 16 years, *p*-value = 3.9E-10; +1.37 sum score at 17 to 28 years, *p*-value = 2.0E-5) and chronic fatigue scores (+0.38 sum score at 15 to 16 years, *p*-value = 0.036; +0.54 sum score at 17 to 28 years, *p*-value = 0.021). Age at assessment was significant for chronic fatigue sum score (−0.55 in sum score per year of age, *p*-value = 0.033) in age group 15 to 16 years, and both the anxiety-depression (−0.17 in sum score per year of age, *p*-value = 0.041) and chronic fatigue sum scores (−0.20 in sum score per year, *p*-value = 0.021) for those aged 17 years and older.

For all the IRT scores, the omnibus test did not reject the null hypothesis of equality of means and variance across groups (Additional file 20). On the other hand, the test returned significant *p*-values (between 0.015 and 3.2E-7) for all but one sum score (chronic fatigue within 15 to 17 age range, *p*-value = 0.59). We winsorised the sum scores to three standard deviations from the mean, in order to limit the influence of extreme values. However, most sum score means and variance were still significantly different across groups (*p*-values in 0.72–4.7E-5, See Additional file 20). In addition, two tests suggested presence of sex limitation, however only on sum scores, and we also attributed these rejections to the skewed distribution. Tests of familial aggregation and presence of genetic effect were significant for all the IRT scores. Non-significant results observed for sum scores in the 17 years and older age group could be attributed to lower power (smallest sample size). Finally, the MZ twin pair correlations were always greater than the DZ correlations suggesting presence of heritability (Additional file 20, Table 6). These results highlight that sum scores are not normally distributed, with overly frequent scores of 0 and a heavy right tail. Winsorisation reduced the weight of extreme observations but did not remove completely the false positives in assumption testing induced by the score distributions.

**Table 6:** Summary of variance component analysis (AE models and twin pair correlations)

	Parameter Estimates	Twin pair correlations		
	A	E	rMZ	rDZ
Anxiety depression				
8–12 years	**0.41 [0.32,0.49]**	**0.59 [0.51,0.68]**	0.43	0.22
13–14 years	0.42 [0.33,0.5]	0.58 [0.5,0.67]	0.38	0.32
15–16 years	0.29 [0.2,0.38]	0.71 [0.62,0.8]	0.28	0.20
17–28 years	**0.37 [0.21,0.51]**	**0.63 [0.49,0.79]**	0.39	0.18
Chronic fatigue				
8–12 years	**0.42 [0.33,0.51]**	**0.58 [0.49,0.67]**	0.42	0.25
13–14 years	**0.51 [0.43,0.59]**	**0.49 [0.41,0.57]**	0.53	0.29
15–16 years	**0.35 [0.25,0.44]**	**0.65 [0.56,0.75]**	0.38	0.15
17–28 years	**0.27 [0.11,0.41]**	**0.73 [0.59,0.89]**	0.27	0.07

Estimates of proportion of variance explained by additive genetics (A) and unique environment (E) calculated from AE models. When AE was the “best model” (i.e. most parsimonious model with no significant difference of fit with full model) the parameters appear in bold. Full tables that include ACE, ADE estimates and model fit comparison are available in Additional file 20

**Fig. 10:**
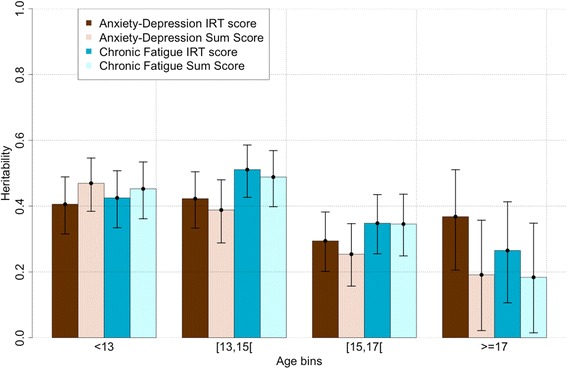
Heritability of anxiety-depression and chronic fatigue scores across age. Bars indicate 95% confidence intervals. Estimates and confidence intervals correspond to the ones from AE models

We fitted ACE and ADE models for IRT and (Winsorised) sum scores in all age groups (see Additional file 20 for summary of model fit). We did not have the power to detect A and C/D simultaneously; due to the modest number of twin-sibling pairs and considering the magnitude of the effects (Additional file 20). In Fig. 10 and Table 6 we report the heritability estimates from an AE model, however, we cannot exclude that a shared environment source of variance may be present for some age groups (Additional file 20). Heritability estimates for anxiety-depression IRT scores were consistent across age groups (h^2^_9-12years_ = 0.41 [0.32,0.49], h^2^_13–14years_ = 0.42 [0.33,0.50], h^2^_15-17years_ = 0.29 [0.20,0.38] and h^2^_17–28years_ = 0.37 [0.21,0.51]) as indicated by overlapping 95% confidence intervals (Fig. 10, Table 6). In each age group, heritability of the sum score (h^2^_9-12years_ = 0.46 [0.37,0.54], h^2^_13–14years_ = 0.40 [0.31,0.49], h^2^_15–16years_ = 0.27 [0.17,0.37] and h^2^_17–28years_ = 0.20 [0.028,0.36]) was comparable to those of the IRT score (Additional file 20).

Heritability of chronic fatigue IRT score was also similar across age group (h^2^_9-12years_ = 0.42 [0.33,0.51], h^2^_13–14years_ = 0.51 [0.43,0.59], h^2^_15–16years_ = 0.35 [0.25,0.44], h^2^_17–28years_ = 0.27 [0.11,0.41]) and consistent with results on sum scores (h^2^_9-12years_ = 0.45 [0.36,0.53], h^2^_13–14years_ = 0.50 [0.41,0.57], h^2^_15–16years_ = 0.36 [0.26,0.45], h^2^_17–28years_ = 0.18 [0.016,0.35]) (Fig. 10, Table 6). Differences between IRT and sum scores could be partially explained by outliers present in the sum score distribution.

The anxiety-depression and chronic fatigue IRT scores were positively correlated (Additional file 20), consistently across age groups (r_9-12years_ = 0.62 [0.58,0.65], r_13–14years_ = 0.67 [0.64,0.70], r_15–16years_ = 0.68 [0.65,0.70] and r_17–28years_ = 0.63 [0.58,0.68]). The phenotypic correlation was mostly driven by the genetic correlation: rG_9-12years_ = 0.87 [0.77,0.98], rG_13–14years_ = 0.85 [0.77,0.98], rG_15–16years_ = 0.88 [0.77,0.90] and rG_17–28years_ = 1.00 [0.88,1.00]. Environmental correlations between anxiety-depression and chronic fatigue were comparatively lower (rE_9-12years_ = 0.44 [0.35,0.52], rE_13–14years_ = 0.52 [0.43,0.59], rE_5-16years_ = 0.58 [0.51,0.64] and rE_17–28years_ = 0.43 [0.31,0.54]). All correlations were significantly different from 0, even after multiple testing correction (*p*-values < 5.2E-7, significance threshold set to 3.0E-4 based on 16 independent tests, Additional file 20). Phenotypic and environmental correlations were also significantly different from 1 (*p*-value < 1.5E-6), suggesting that anxiety-depression and chronic fatigue only share a fraction of their environmental sources of variance. Correlations between the sum scores differed little in strength and supported the same conclusions (Additional file 20). Finally, the genetic correlations were only significantly different from 1 at ages 15 to 16 years for the IRT score (*p*-value = 7.0E-4) and before age 15 for sum scores (*p*-values < 3.1E-6) suggesting that most of the genetic sources of variance are common to the two SPHERE-21 scores (Additional file 20). We investigated the impact on the correlations of the three items common to both scales, by removing them from the anxiety-depression score. Their exclusion had little impact on the genetic correlations (rG_9-12years_ = 0.83 [0.71,0.95], rG_13–14years_ = 0.87 [0.77,0.99], rG_15–16years_ = 0.91 [0.75,1.00] and rG_17–28years_ = 0.97 [0.75,1.00]) and did not change the conclusions reported above (see Additional file 20 for all correlations and *p*-values).

Previous results on the total sample (1168 complete pairs aged 12 to 25 years, [6]) reported similar heritabilities around 0.40 as well as correlations (*r* = 0.60, rG = 0.87 and rE = 0.41) between anxiety-depression and somatisation sum scores. Here, we expanded these results by showing consistent heritability and correlation between SPHERE-21 scores in different age groups. Results can be compared across publications as we used the same definition for the anxiety-depression scale, and combined the somatisation and fatigue scales that showed almost perfect genetic correlations (Additional file 1).

### SPHERE-21 association with some DSM-IV psychiatric diagnoses

**Fig. 11:**
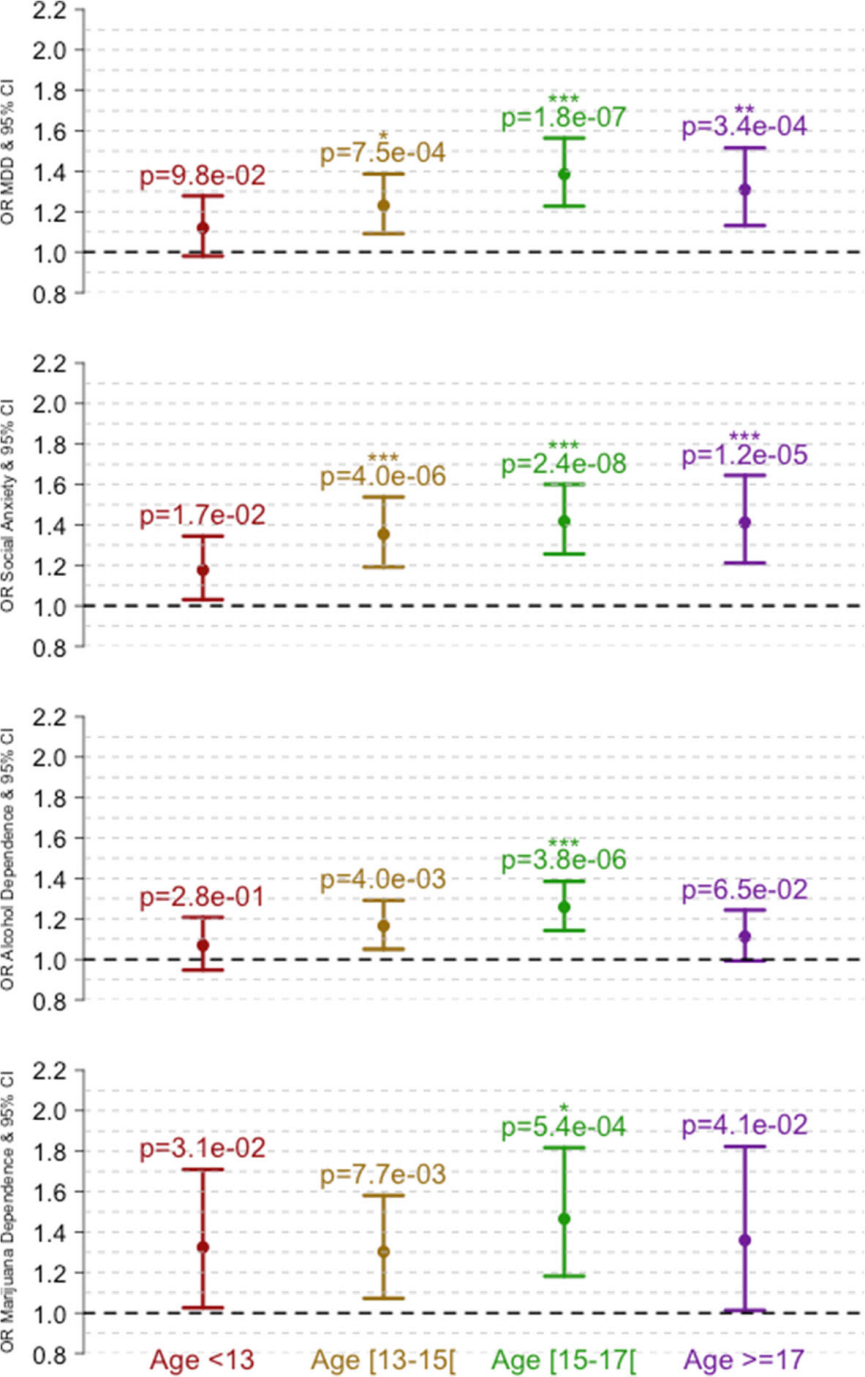
Risks of MDD, social anxiety and substance dependence increases with anxiety-depression IRT scores. *p*-values are indicated above 95% confidence intervals. The *stars* correspond to significance after correcting for multiple testing (Bonferonni correction). *corresponds to p_corrected_ < 0.05, **p_corrected_ < 0.01 and ***p_corrected_ < 0.001

We tested the association of SPHERE-21 scores from earlier ages with DSM-IV diagnoses (MDD, social anxiety, alcohol and marijuana dependence) assessed with the CIDI after age 19 (mean age 22). We estimated the number of independent SPHERE-21 scores to be six [78], yielding a significance threshold of 2.1E-3 corresponding to an estimated 24 independent tests. The anxiety-depression IRT scores were associated with increased MDD risk (OR_13–15_ = 1.23 [1.09,1.39], *p* = 7.4E-4; OR_15–16_ = 1.39 [1.22,1.56], *p* = 1.8E-7; OR_17–28_ = 1.31 [1.13,1.52], *p* = 3.4E-4), as well as increased risk of social anxiety (OR_13–14_ = 1.35 [1.19,1.54], *p* = 3.9E-6; OR_15–16_ = 1.42 [1.26,1.60], *p* = 2.4E-8 and OR_17–28_ = 1.41 [1.21,1.65], *p* = 1.2E-5), alcohol dependence (OR_15–16_ = 1.26 [1.14,1.39], *p* = 3.8E-6) and marijuana dependence (OR_15–16_ = 1.47 [1.18,1.82], *p* = 5.3E-4). All other odds ratios were greater than 1 but did not reach significance (Fig. 11).

**Fig. 12:**
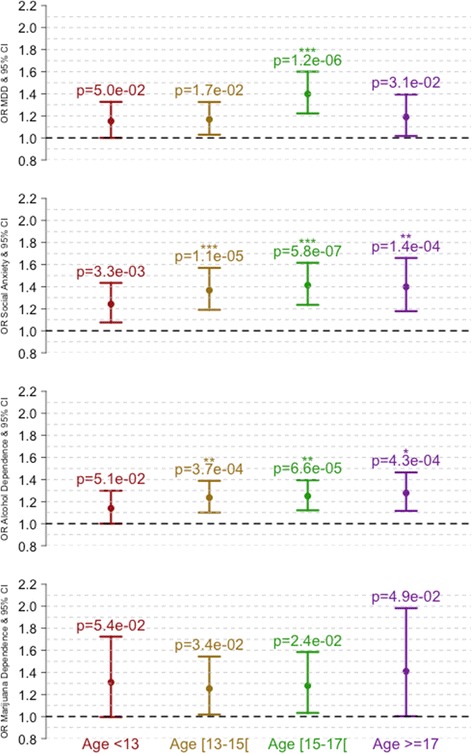
Risks of MDD, social anxiety and substance dependence increases with chronic fatigue IRT scores. *p*-values are indicated above 95% confidence intervals. The stars correspond to significance after correcting for multiple testing (Bonferonni correction). *corresponds to p_corrected_ < 0.05, **p_corrected_ < 0.01 and ***p_corrected_ < 0.001

Chronic fatigue IRT scores were also associated with increased risk of MDD (OR_15–16_ = 1.39 [1.22, 1.60], *p* = 1.2E-6), social anxiety (OR_13–14_ = 1.38 [1.19, 1.57], *p* = 1.1E-5; OR_15–16_ = 1.41 [1.23, 1.62], *p* = 5.8E-7 and OR_17–28_ = 1.40 [1.18, 1.66], *p* = 1.4E-4) and alcohol dependence (OR_13–14_ = 1.24 [1.10, 1.39], *p* = 3.7E-4; OR_15–16_ = 1.25 [1.12, 1.39], *p* = 6.6E-5 and OR_17–28_ = 1.28 [1.12, 1.46], *p* = 4.3E-4) (Fig. 12). Such odds ratios (1.09 to 1.82 from the confidence intervals) translate to a 0.6 to 6 fold increased risk between individuals with minimal (−3) and maximal (+3) IRT score.

Sum scores showed the same pattern of association, except for anxiety-depression in those aged 17 to 28 years, which did not reach significance (*p* = 7.3E-3, Additional file 21 and Additional file 22). Effect sizes were comparable, taking into account the difference in range between IRT and sum scores (Additional file 21 and Additional file 22).

### Comparison of SPHERE-21 and Beck’s depression inventory

Compared with the psychometric properties of the “gold standard” Beck Depression Inventory (BDI-II) [80, 81], the SPHERE-21 is considerably shorter for measuring anxiety-depression (14 vs. 21 items) and provides an additional measurement of chronic fatigue. While studies on the latent structure of the BDI consistently identify two dimensions: cognitive-affective and somatic-vegetative [81], more sophisticated modelling showed that much of the variance of the BDI could be explained by a general construct, and BDI subscales are rarely used in practice [81]. Furthermore, combining cognitive-affective and somatic-vegetative symptoms may be appealing as it matches the DSM-IV (and DSM-5) definition of MDD. Based on our results, the high genetic correlation between anxiety-depression and chronic fatigue could justify combining the two scales, as the same genetic factors would contribute to both traits. However, anxiety-depression and chronic fatigue shared less than half of their environmental sources of variance and separating them in analyses could help identify specific environmental contributors [82].

Psychometric properties of the BDI have been studied for more than 50 years [80, 81]. However, most of the early studies suffered from lack of powerful statistical methods (such as IRT). Based on omnibus measures of test-retest and internal consistency, the BDI shows very good psychometric properties, comparable to SPHERE-21 (Table 7). However, more in depth assessments [53] revealed that two items of the BDI (9 “Suicidal wishes” and 10 “Crying”) failed to meet IRT requirements of monotonicity of IRSF in depressed outpatients and non-patient college students [53], potentially leading to bias in score and misordering of the participants on the sum score. In addition, item 19 (“Weight loss”) correlated poorly with the latent trait, thus not contributing to the scale and potentially breaching unidimensionality [53]. Finally, item 14 (“Distortion of image body”) showed large sex DIF (DIF = 0.32), being endorsed more often by women [53]. These do not invalidate the BDI, as it has also been shown to effectively measure depression in both clinical and non-clinical settings, and across different languages and populations [81]. However, one can question what impact score bias, sex differential functioning, and participants’ misordering have on a study’s power and predictive ability.

**Table 7:** Comparative psychometric properties of the SPHERE-21 and BDI

	SPHERE-21	BDI
Number of items	14 (for anxiety-depression)	21
Short form for clinical use	SPHERE-12 (six items for anxiety-depression)	BDI-11
IRT requirements	Monotonicity verified Good correlation of items with the latent trait	Monotonicity breached for items 9 and 10 Poor correlation of item 19 with the latent trait [53]
Sex DIF	Limited (DIF < 0.25)	Large DIF for item 14 (DIF = 0.32) Limited otherwise (DIF < 0.25) [53]
Age group DIF	Limited (DIF < 0.25); comparable construct from age 9 to 28 years	Not tested at item level (IRT) Not investigated in a population sample Comparable structure and internal consistency (Cronbach’s alpha) for adolescent inpatients [98]
Language(s)	Arabic, Cantonese, Croatian, Dutch, English, Greek, Italian, Japanese, Mandarin, Portuguese, Serbian, Spanish, Turkish, Vietnamese^a^	Arabic, Chinese, English, Farsi, Finnish, French, German, Japanese, Korean, Norwegian, Portuguese, Spanish, Swedish, Turkish
Test-retest	0.47 [0.23,0.66] at three months	0.48–0.86 [80] depending on the sample and test-retest interval
Cronbach’s alpha	0.87	0.81 [80, 81]
Heritability	0.41 [0.32,0.49] between ages nine and 12 years, 0.42 [0.33,0.50] at 13 to 14 years, 0.29 [0.20,0.38] at 15 to 16 years and 0.37 [0.21,0.51] between ages 17 and 28 years (AE models, anxiety-depression scores)	0.18 [0.05,0.31] (AE model, mean age 31 years, range 16–71) [84]
Association with DSM-IV diagnoses	Significant from age 15 years with alcohol and Marijuana dependence; and from age 13 years for MDD and social anxiety (anxiety-depression subscale).	Not evaluated in general population
Price	Free	Around 2 USD per questionnaire [99]

^a^Questionnaires in non-English languages available on demand. Please contact Pr. Ian Hickie (ian.hickie@sydney.edu.au)

Here, we used the BDI questionnaire as gold standard as it is one of the oldest, most used and most tested depression questionnaire. For other widely used questionnaires such as the Achenbach or Hamilton rating scales, some the methods used here (e.g. NIRT modelling, twin models) have never been applied, which makes the comparison less meaningful

Heritability of the BDI score has been reported from large family data (*N* = 200 from 12 families) [83] or broken down into subscales (343 twin pairs) [84]. The first study reported heritabilities between 0.45 and 0.87, while the second could not conclude regarding the presence of heritability or common environment factors, explaining 2 to 30% of the score variance. Larger twin studies are required to provide more accurate heritability estimates of the BDI across ages.

Finally, the BDI has been evaluated many times as a prediction tool for MDD in clinical settings [81]. A few studies have focused on non-clinical samples but suffered several limitations: a) small samples; b) samples with greater prevalence than in general population; c) non-DSM-based diagnoses; and mostly, d) use of score cut-off criteria which defeats the purpose of using a continuous score but also makes comparison of specificity and sensitivity impossible across studies when different cut-offs are used (see [81] for a review of these studies). We could not find a publication reporting the association between the BDI score and disease risk in the general population, and much testing remains to be done on the BDI to validate its use in population samples and non-clinical research. Comparison of SPHERE-21 and BDI qualities is summarised in Table 7.

There are several limitations to the SPHERE-21 that are worth mentioning – it has only been tested in an Australian-based population sample of young people, and previously on clinical participants [1, 85–89]. Thus, more testing and DIF investigation is required on older participants, patients with specific pathologies or different cultures and ethnic groups. Use of the SPHERE-21 in other English-speaking countries may require some items to be reworded. For example, for item 2, the word “cranky”, not frequently used in the United States, could be replaced by “easily irritated”. The scalability of the BDI across countries and languages led to its world-wide popularity [81], though only recently was IRT used [90–94], and little has been done to assess cross-cultural comparability of the BDI scale [81] (e.g. DIF by culture or ethnicity). In addition, unlike the BDI [81], correlations between the SPHERE-21 scores with other measures of anxiety, depression or fatigue remains to be investigated. The only published research showed a positive correlation (and significant genetic relationship) of the anxiety-depression sum scores with neuroticism [6]. SPHERE-34 was also shown to have some value in screening for psychiatric morbidity [89]. Finally, the SPHERE-21 lacks positive item results in a skewed distribution, but this limitation also applies to the BDI [81]. A simple way to improve the score distribution may be to separate options “never” and “sometimes” during SPHERE-21 questionnaire collection, as it may provide more information about individuals with low anxiety-depression and fatigue (Additional file 23).

## Conclusions

Here, we examined the use of the SPHERE-34 for assessment of anxiety, depression and fatigue in a large Australian-based population sample of young people. Using an NIRT analysis we showed that the questionnaire could be reduced to 21 items (SPHERE-21), providing a measure of anxiety-depression (14 items) and chronic fatigue (10 items). We showed that these two scales of the SPHERE-21 measured valid and comparable constructs across sexes and age groups (from age 9 to 28 years), and that both showed moderate reliability, high internal consistency and good item scalability. We also showed that the SPHERE-21 scores were moderately heritable and genetically correlated across adolescence, correlation that was not due to the items common to both scales. In addition, we showed that anxiety-depression and chronic fatigue were, from an early age (13 or 15 years) significantly associated with a later risk of MDD, social anxiety and alcohol dependence. This further validates the SPHERE-21 by demonstrating its predictive ability in the general population and its relevance to measure anxiety-depression and chronic fatigue across adolescence and into adulthood. Finally, in a post-hoc evaluation, we suggest that the psychometric properties of the SPHERE-21, are at least equivalent to those of the Beck Depression Inventory, in an Australian-based population sample of young people.

## Additional files

Additional file 1: Correlation between fatigue and somatisation scales from the SPHERE-34 sum scores. (DOCX 60 kb)

Additional file 2: Treatment of missing values. (DOCX 112 kb)

Additional file 3: Response Step Function of the 15 items proposed to measure chronic fatigue. (PNG 1115 kb)

Additional file 4: Response Step Function of the 9 items proposed to measure chronic fatigue after exclusion of items not meeting IRT requirements. (PNG 743 kb)

Additional file 5: Sex DIF for the 14 items of the anxiety-depression scale (TA wave). (PNG 1741 kb)

Additional file 6: Sex DIF for the 14 items of the anxiety-depression scale (TM wave). (PNG 1702 kb)

Additional file 7: Sex DIF for the 14 items of the anxiety-depression scale (TW2 wave). (PNG 1700 kb)

Additional file 8: Sex DIF for the 14 items of the anxiety-depression scale (TW1 wave). (PNG 1715 kb)

Additional file 9: DIF between studies TM and TA (anxiety-depression scale). (PNG 1696 kb)

Additional file 10: DIF between studies TW2 and TA (anxiety-depression scale). (PNG 1711 kb)

Additional file 11: DIF between studies TW1 and TA (anxiety-depression scale). (PNG 1714 kb)

Additional file 12: Sex DIF for the 11 items of the chronic fatigue scale (TA wave). (PNG 1376 kb)

Additional file 13: Sex DIF for the 11 items of the chronic fatigue scale (TM wave). (PNG 1339 kb)

Additional file 14: Sex DIF for the 11 items of the chronic fatigue scale (TW2 wave). (PNG 1358 kb)

Additional file 15: Sex DIF for the 11 items of the chronic fatigue scale (TW1 wave). (PNG 1370 kb)

Additional file 16: DIF between studies TM and TA (chronic fatigue scale). (PNG 1342 kb)

Additional file 17: DIF between studies TW2 and TA (chronic fatigue scale). (PNG 1330 kb)

Additional file 18: DIF between studies TW1 and TA (chronic fatigue scale). (PNG 1350 kb)

Additional file 19: SPHERE-21. (DOCX 22 kb)

Additional file 20: Summary of genetic analyses: sampling homogeneity testing, ACE/ADE estimates and model fit comparison. (DOCX 197 kb)

Additional file 21: Risk of MDD, social anxiety and substance dependence increases with anxiety-depression sum scores. (PNG 76 kb)

Additional file 22: Risk of MDD, social anxiety and substance dependence increases with chronic-fatigue sum scores. (PNG 77 kb)

Additional file 23: Glossary. (DOCX 92.5 kb)

## Abbreviations

BDI: Beck’s Depression Inventory
IRSF: Item Response Step Function
IRT: Item Response Theory
MSA: Mokken Scale Analysis
NIRT: Non-parametric IRT
PIRT: Parametric IRT
SPHERE: Somatic and Psychological HEalth Report
SPHERE-12: Short version of the SPHERE-34 questionnaire, limited to 2 scales of 6 items often referred to as psychological distress and somatic distress. This version was originally created for screening purposes in general practice [1]
SPHERE-21: New reduced version of the SPHERE-34 questionnaire comprising of 2 scales: anxiety-depression (14 items) and chronic fatigue (10 items) with 3 overlapping items. These modified scales show good psychometric properties and can be safely used to study changes through adolescence in the general population
SPHERE-34: Full SPHERE questionnaire that comprises 34 items, with several scales calculable: anxiety-depression, fatigue, somatisation and neurasthenia (deprecated)
TA: Twin adolescent study
TM: Twin memory, attention and problem solving study
TW1: Twin mole study visit 1
TW2: Twin mole study visit 2
