# Supplementary material for: Validation and psychometric properties of the Somatic and Psychological HEalth REport (SPHERE) in a young Australian-based population sample using non-parametric item response theory
Source: BMC Psychiatry. 2017 Aug 1;17:279. doi: 10.1186/s12888-017-1420-1 (PMC5540428; doi:10.1186/s12888-017-1420-1)
Supplement: Supplementary file 1 — Correlation between fatigue and somatisation scales from the SPHERE-34 sum scores. (DOCX 60 kb) [file 12888_2017_1420_MOESM1_ESM.docx]

Correlation between Fatigue and Somatisation scales (SPHERE-34 sum scores**)**

|  | **Phenotypic correlation  [95% CI]** | **Genetic correlation [95% CI]** | **Environmental correlation [95% CI]** |
| --- | --- | --- | --- |
| **TW1** | 0.88 [0.86, 0.89] | 0.97 [0.94,1.00] | 0.80 [0.76,0.83] |
| **TW2** | 0.90 [0.98,0.91] | 0.97 [0.95,1.00] | 0.84 [0.81,0.870.9] |
| **TM** | 0.92 [0.91,0.93] | 0.98 [0.96,1.00] | 0.88 [0.86,0.90] |
| **TA** | 0.91 [0.90,0.92] | 0.97 [0.93,1.00] | 0.88 [0.85,0.91] |

Supplementary Table 1: Phenotypic, genetic and environmental correlations between Fatigue and Somatisation subscales of the SPHERE-34 questionnaire

Correlations were estimated in OpenMx, see main manuscript for sample sizes.
