# Supplementary material for: Validation and psychometric properties of the Somatic and Psychological HEalth REport (SPHERE) in a young Australian-based population sample using non-parametric item response theory
Source: BMC Psychiatry. 2017 Aug 1;17:279. doi: 10.1186/s12888-017-1420-1 (PMC5540428; doi:10.1186/s12888-017-1420-1)
Supplement: Supplementary file 2 — Treatment of missing values. (DOCX 112 kb) [file 12888_2017_1420_MOESM2_ESM.docx]

### Treatment of missing values

We reported the sample missingness per individuals, twin pairs and items (Table 1). In addition, we compared the age, sex and SPHERE-34 sum scores between missing and non-missing groups to investigate whether data was missing (completely) at random (MCAR or MAR). MCAR or MAR (i.e. dependent on covariates such as age or sex but independent of the outcome variables) is necessary to ensure unbiased results after exclusion of missing values or imputation [[42](#_ENREF_42)].

Overall, the number of missing values was fairly low in the studies. In TA, comprising the most missing observations, only 4% of individuals had one or more missing item, with 3 individuals (0.24%) having more than 5 items missing (Table 1). Missingness was not localised to one particular item as shown by the maximal percentage of missing observations across all 34 items (0.63%). Missingness was even rarer in TW1, TW2 studies with no individual having more than 5 items missing. The TM study was even exempt from missing values.

In order to study the pattern of missingness, we compared individuals with one or more item missing with individuals who fully completed the questionnaire. We compared the groups in term of age, sex, frequency, and the original SPHERE summed score (Fisher exact test and Mann-Whitney-Wilcoxon test). Two tests returned a p-value of 0.03, which can only be interpreted as a suggestive difference between groups as such result can be expected by chance, knowing that 21 tests were performed. At a young age (i.e. mean age 12), missingness could be associated with larger levels of somatic distress (median score 4 vs 2) and fatigue (median score 3 vs 2). This effect, if true, is specific to a class age as it was not observed in following studies at later age.

In the following, we assumed missingness to be completely at random as it was not significantly associated with demographic variables or SPHERE scores. Thus, we can be more confident that the exclusion of the 70 individuals with missing values does not induce a selection bias in the analysis. In addition, the impact on power of such exclusion should be limited.

|  | | **TW1** | **TW2** | **TM** | **TA** |
| --- | --- | --- | --- | --- | --- |
| **N**  **Mean age [range] (yrs)** | | 1,707  12 [9-18] | 1,273  14 [9-18] | 1,513  16 [15-22] | 1,264  18 [11-28] |
| **% (N) indiv with >0 missing value** | | 0.70% (12) | 0.63% (8) | 0% | 3.96% (50) |
| **% (N) indiv with >5 missing values** | | 0% | 0% | 0% | 0.24% (3) |
| **maximal % (N) missingness per item** | | 0.12% (2) | 0.16% (2) | 0% | 0.63% (8) |
| **Test of predictors of missingness** (p-values) | **Age** | 0.59 | 0.37 | NA | 0.86 |
|  | **Sex** | 0.15 | 0.73 | NA | 0.11 |
|  | **Depression-Anxiety (SPHERE 12)** | 0.17 | 0.11 | NA | 0.16 |
|  | **Chronic Fatigue (SPHERE 12)** | **0.031** | 0.40 | NA | 0.84 |
|  | **Depression-Anxiety (SPHERE 34)** | 0.055 | 0.21 | NA | 0.11 |
|  | **Somatic-Distress (SPHERE 34)** | 0.053 | 0.93 | NA | 0.60 |
|  | **Fatigue (SPHERE 34)** | **0.031** | 0.42 | NA | 0.93 |

Additional Table 1: Missingness at each time point and investigation of MCAR and MAR hypotheses
When testing sex ratio differences, Fisher exact test were used instead of chi-2 test to overcome the issue of small effectives. For the same reason, we preferred the Mann-Whitney-Wilcoxon test to the Student t-test to compare age and sum scores. Study TM does not have missing values as it was collected in the clinics with the research assistant making sure not question was omitted. Missingness rate is larger in study TA as data was collected at home. It is the only collection time point that was not conducted in the clinics.
