# Supplementary figures and images for: Validation and psychometric properties of the Somatic and Psychological HEalth REport (SPHERE) in a young Australian-based population sample using non-parametric item response theory
Source: BMC Psychiatry. 2017 Aug 1;17:279. doi: 10.1186/s12888-017-1420-1 (PMC5540428; doi:10.1186/s12888-017-1420-1)

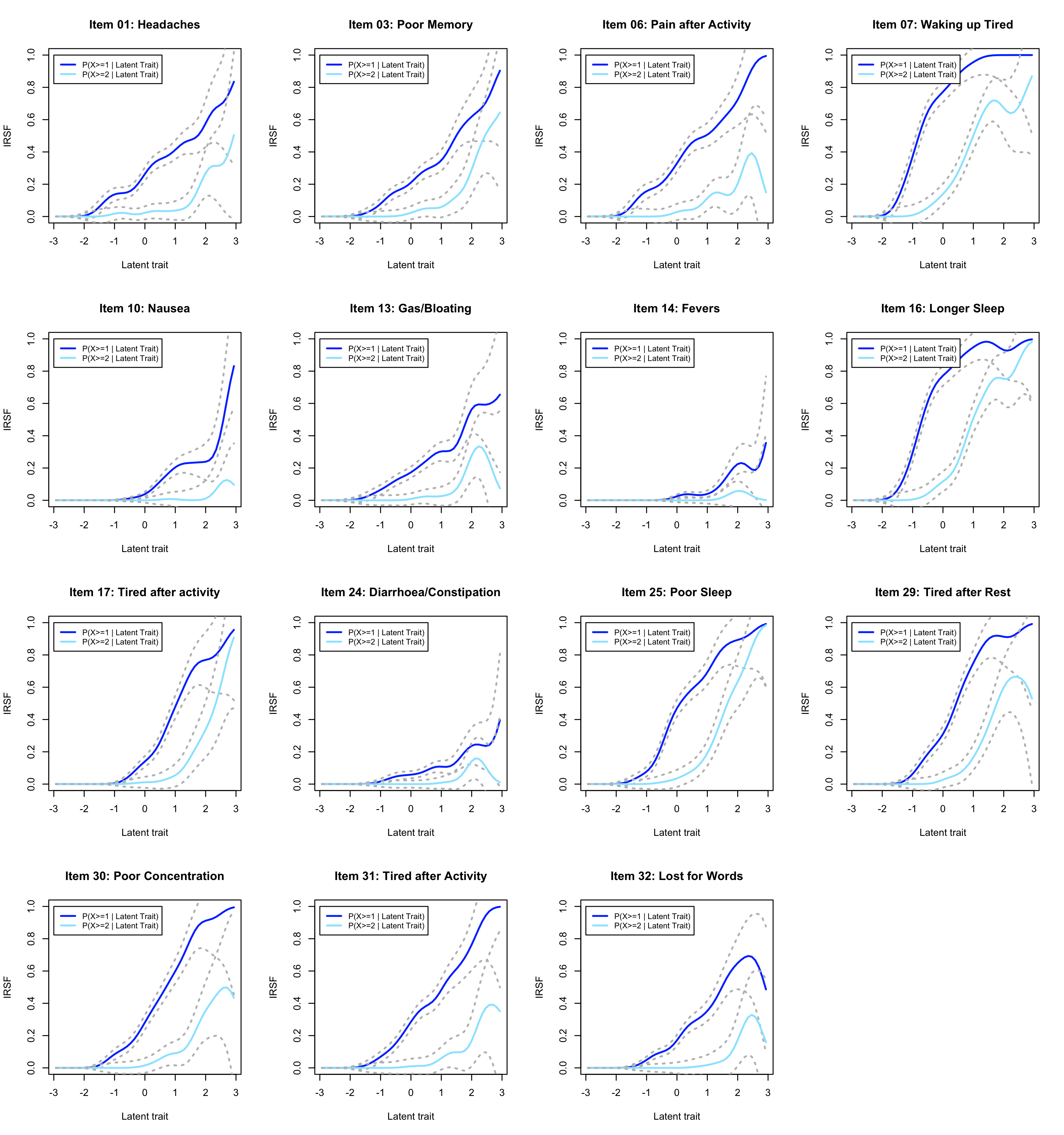

Supplement: Supplementary file 3 — Response Step Function of the 15 items proposed to measure chronic fatigue. (PNG 1115 kb) [file 12888_2017_1420_MOESM3_ESM.png]

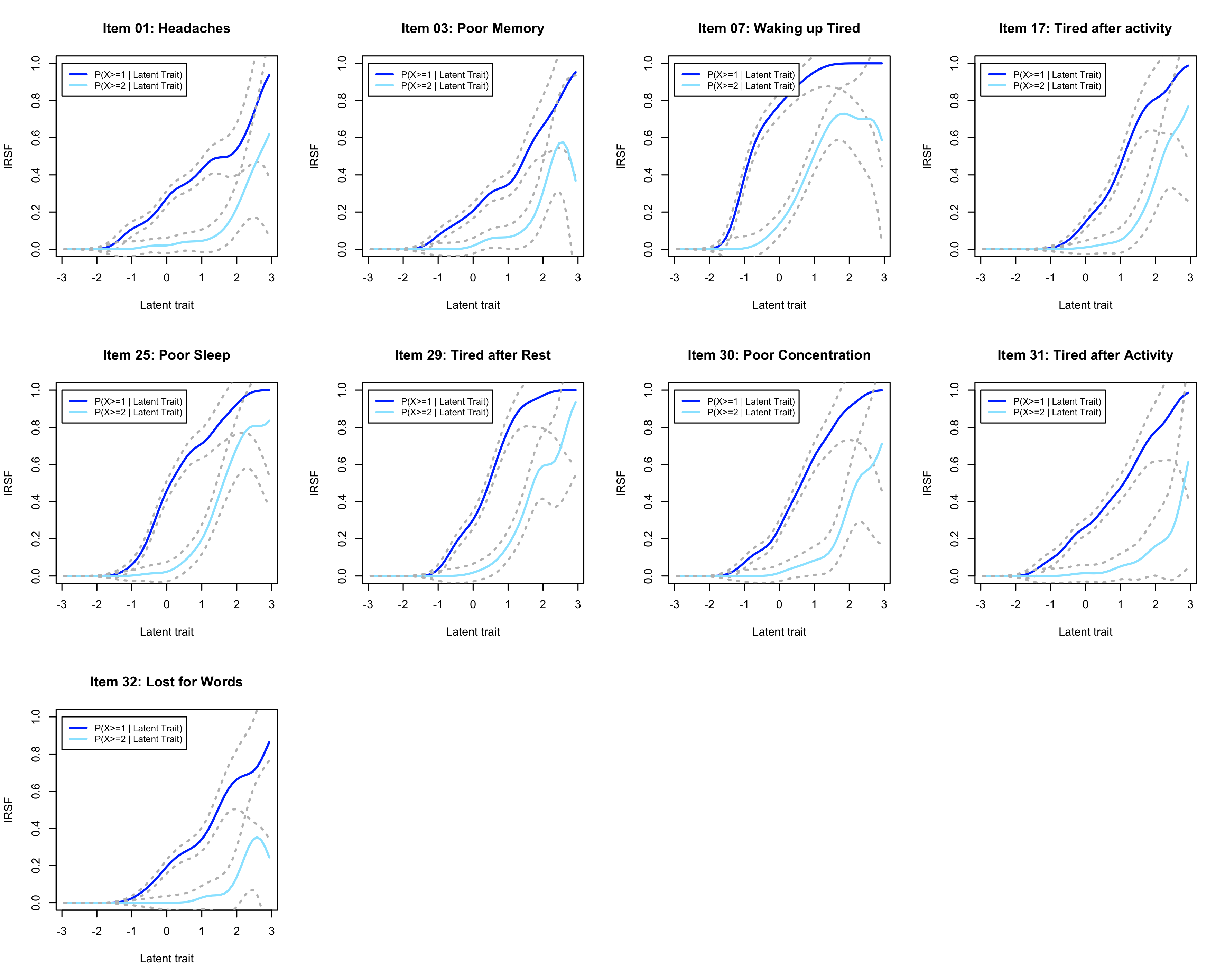

Supplement: Supplementary file 4 — Response Step Function of the 9 items proposed to measure chronic fatigue after exclusion of items not meeting IRT requirements. (PNG 743 kb) [file 12888_2017_1420_MOESM4_ESM.png]

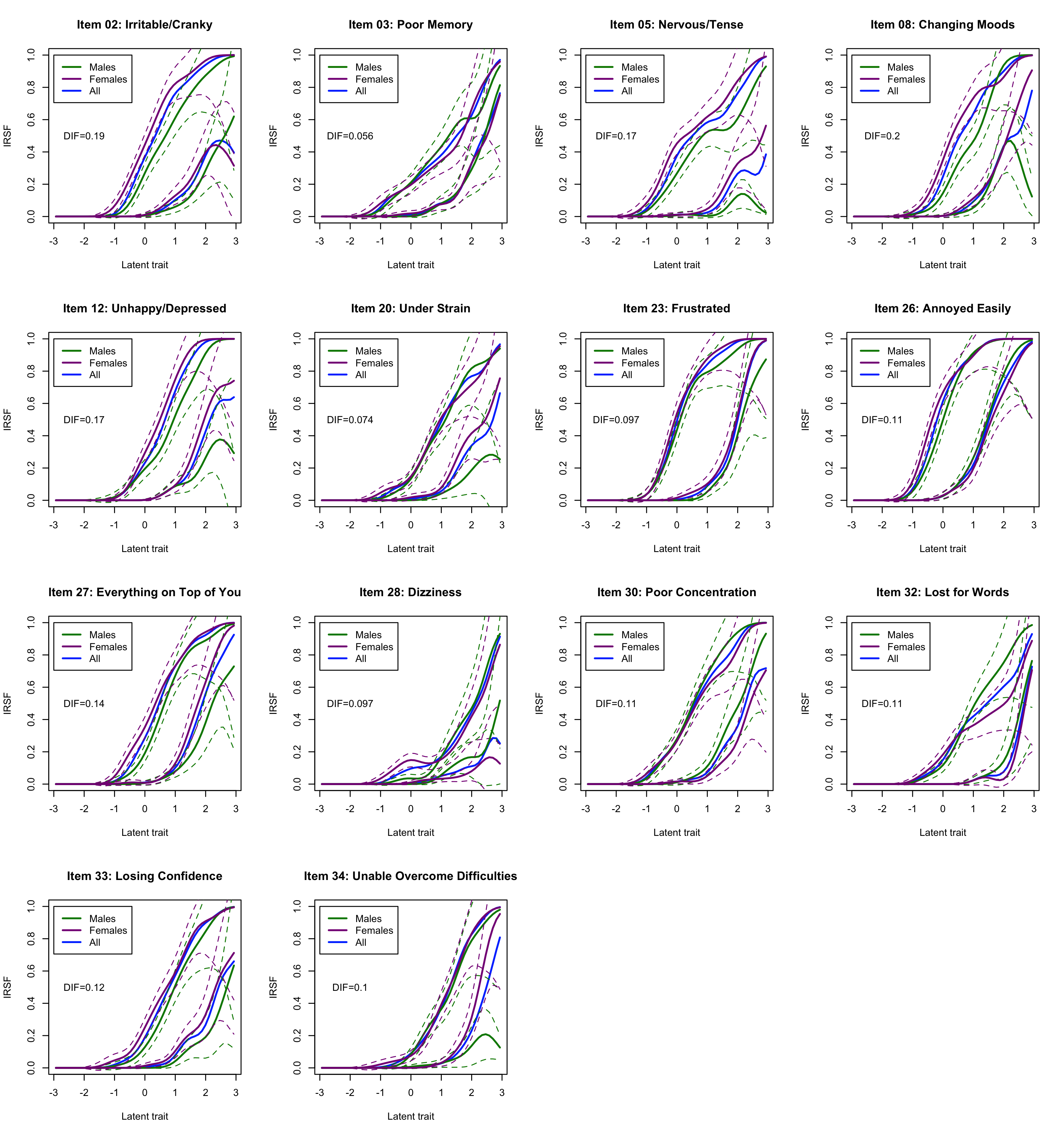

Supplement: Supplementary file 5 — Sex DIF for the 14 items of the anxiety-depression scale (TA wave). (PNG 1741 kb) [file 12888_2017_1420_MOESM5_ESM.png]

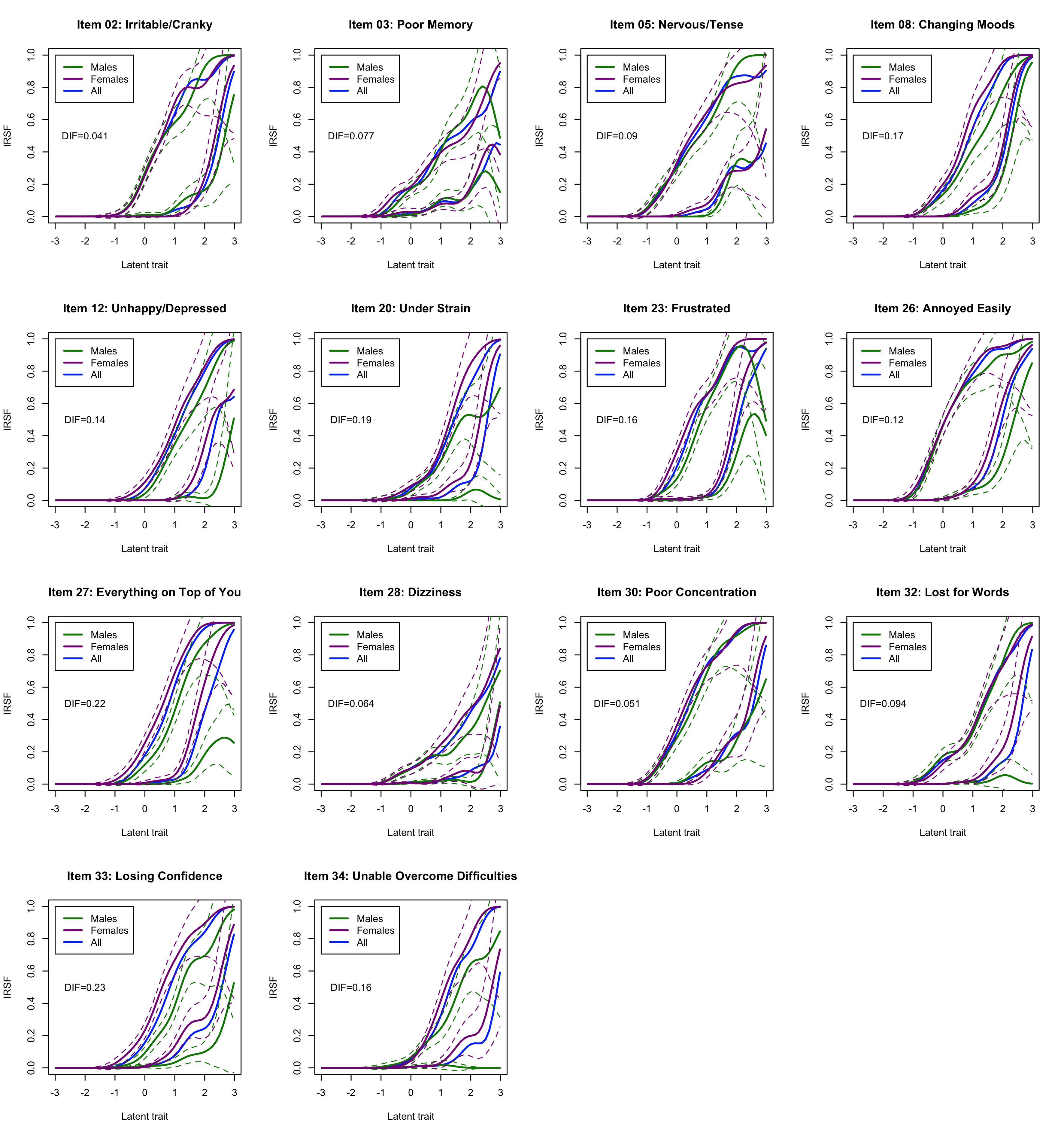

Supplement: Supplementary file 6 — Sex DIF for the 14 items of the anxiety-depression scale (TM wave). (PNG 1702 kb) [file 12888_2017_1420_MOESM6_ESM.png]

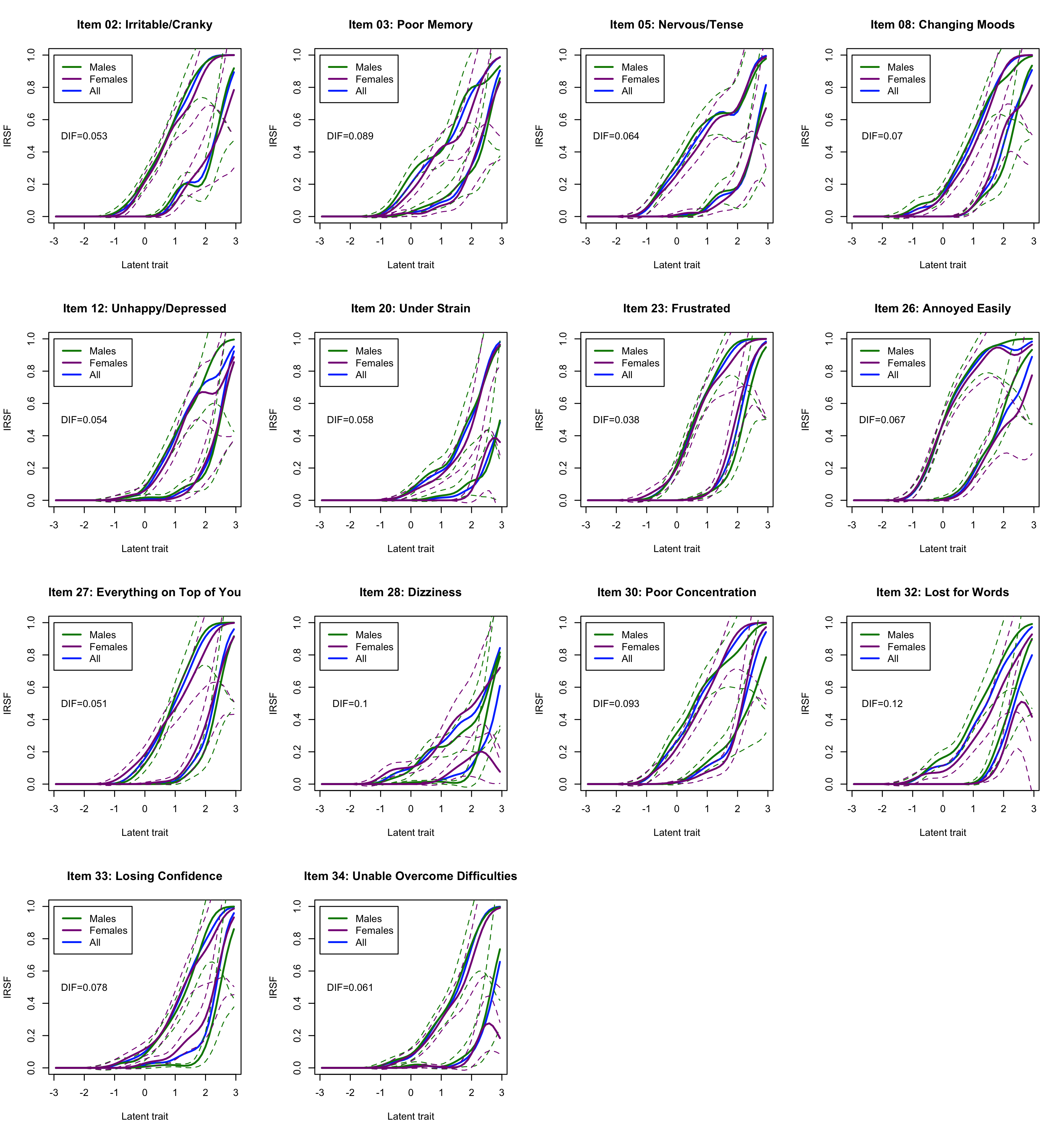

Supplement: Supplementary file 7 — Sex DIF for the 14 items of the anxiety-depression scale (TW2 wave). (PNG 1700 kb) [file 12888_2017_1420_MOESM7_ESM.png]

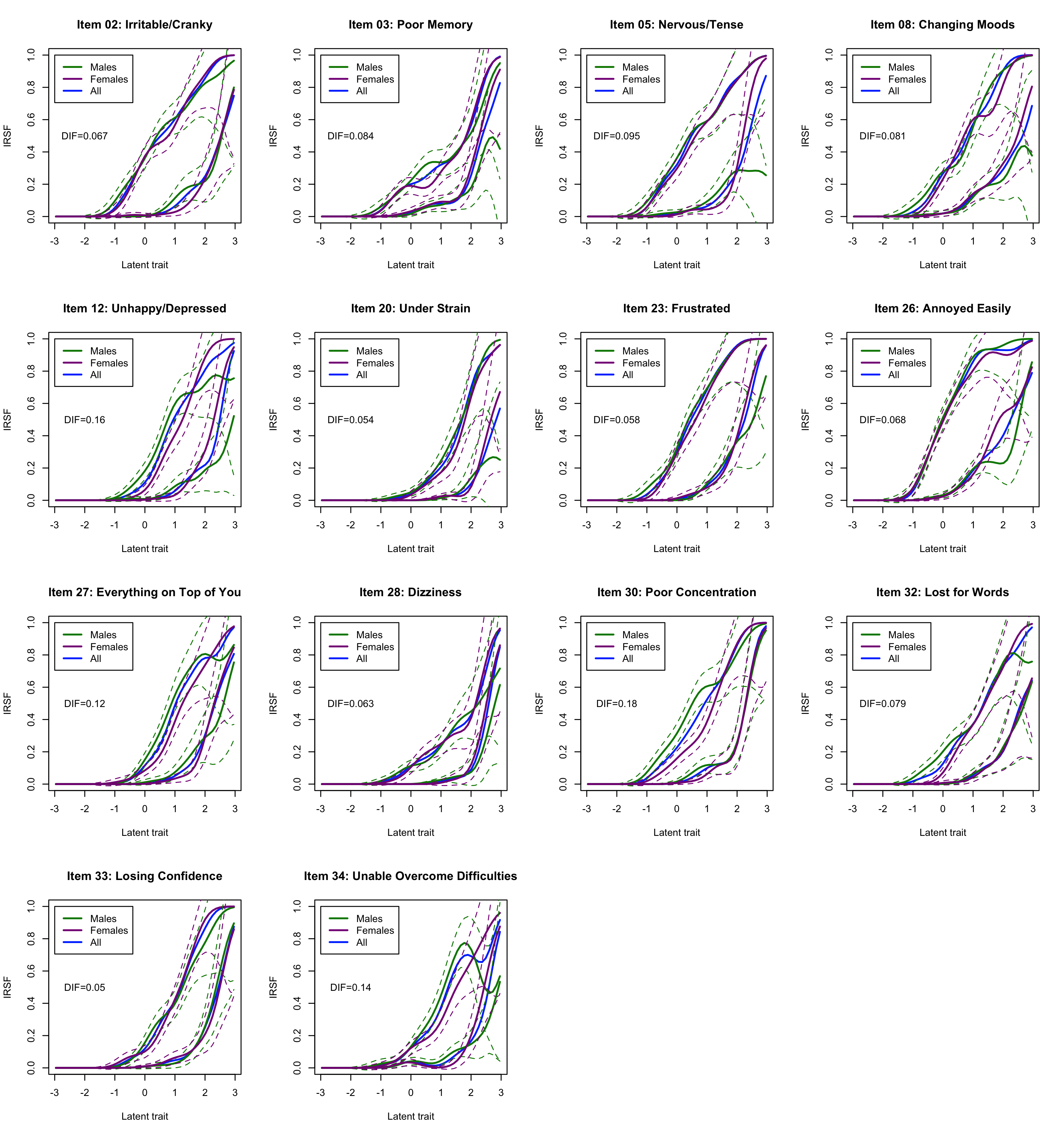

Supplement: Supplementary file 8 — Sex DIF for the 14 items of the anxiety-depression scale (TW1 wave). (PNG 1715 kb) [file 12888_2017_1420_MOESM8_ESM.png]

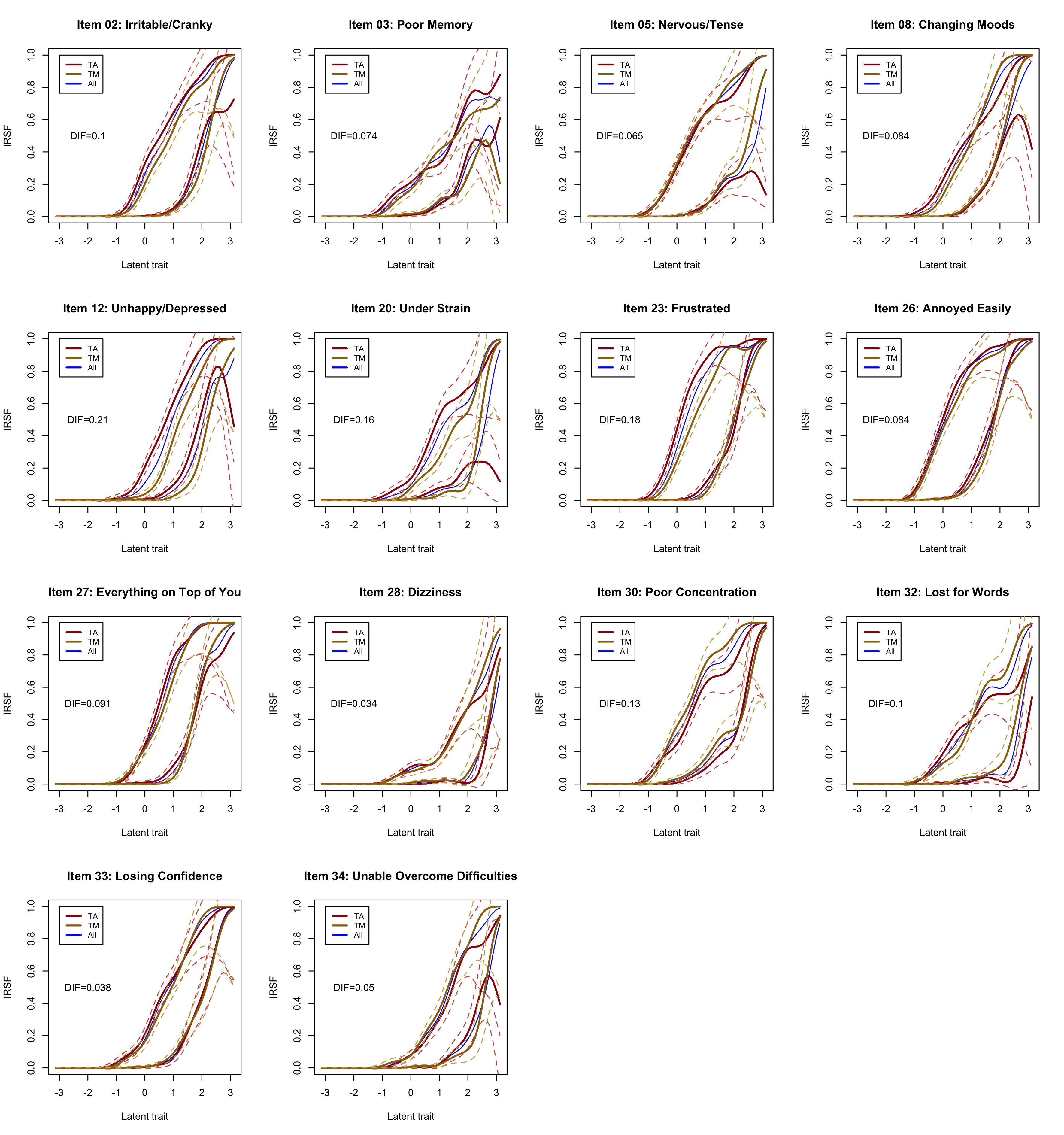

Supplement: Supplementary file 9 — DIF between studies TM and TA (anxiety-depression scale). (PNG 1696 kb) [file 12888_2017_1420_MOESM9_ESM.png]

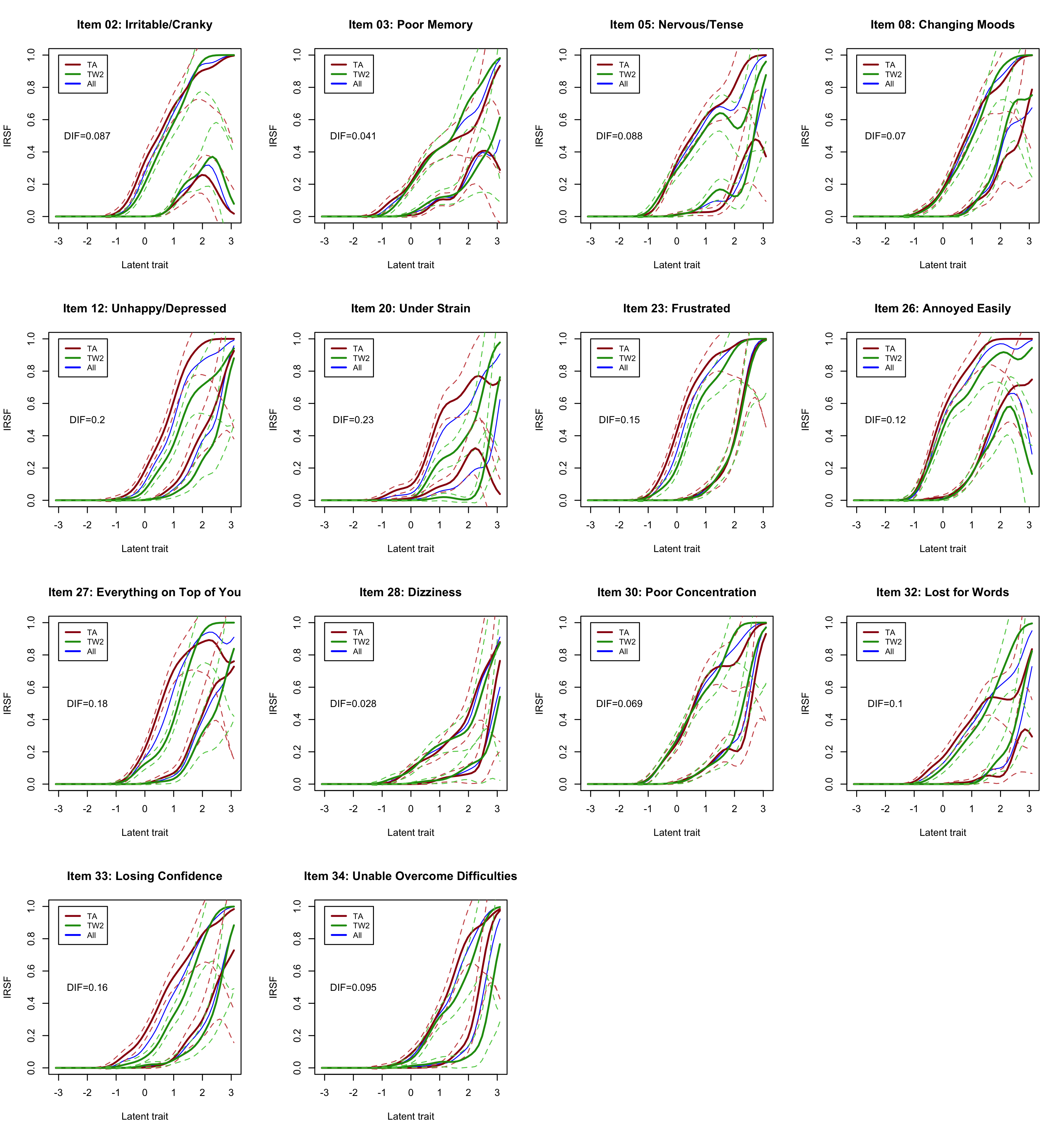

Supplement: Supplementary file 10 — DIF between studies TW2 and TA (anxiety-depression scale). (PNG 1711 kb) [file 12888_2017_1420_MOESM10_ESM.png]

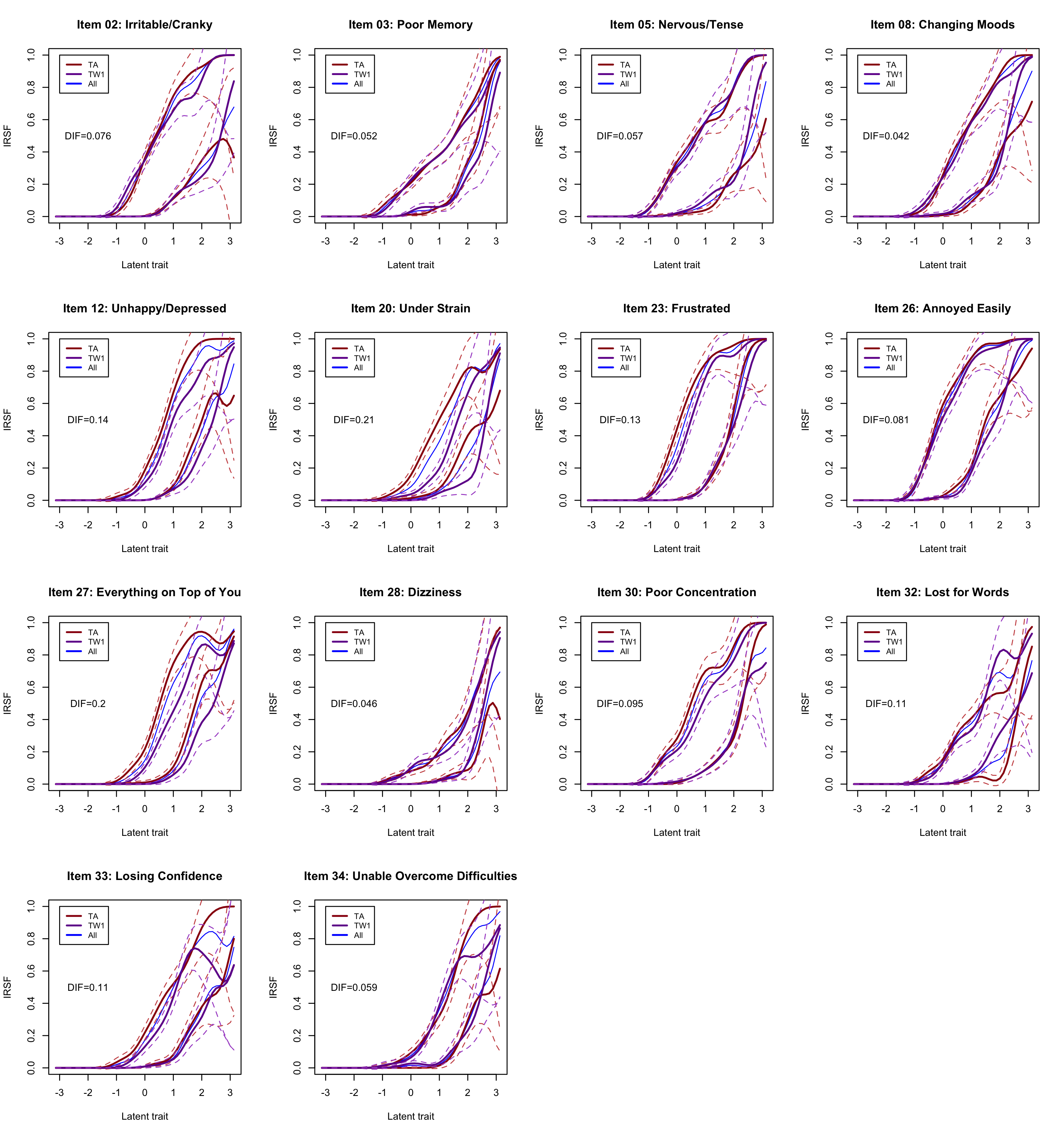

Supplement: Supplementary file 11 — DIF between studies TW1 and TA (anxiety-depression scale). (PNG 1714 kb) [file 12888_2017_1420_MOESM11_ESM.png]

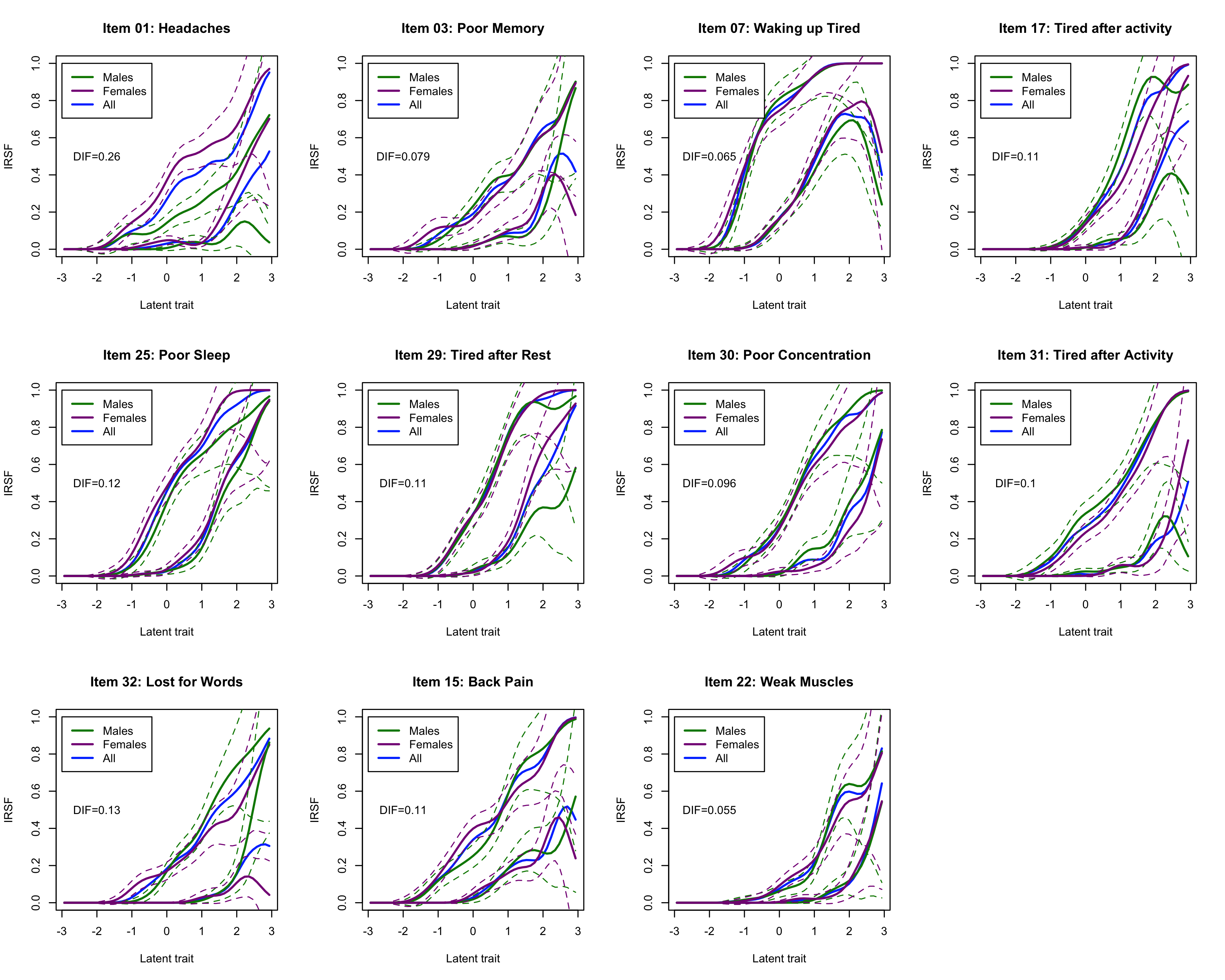

Supplement: Supplementary file 12 — Sex DIF for the 11 items of the chronic fatigue scale (TA wave). (PNG 1376 kb) [file 12888_2017_1420_MOESM12_ESM.png]

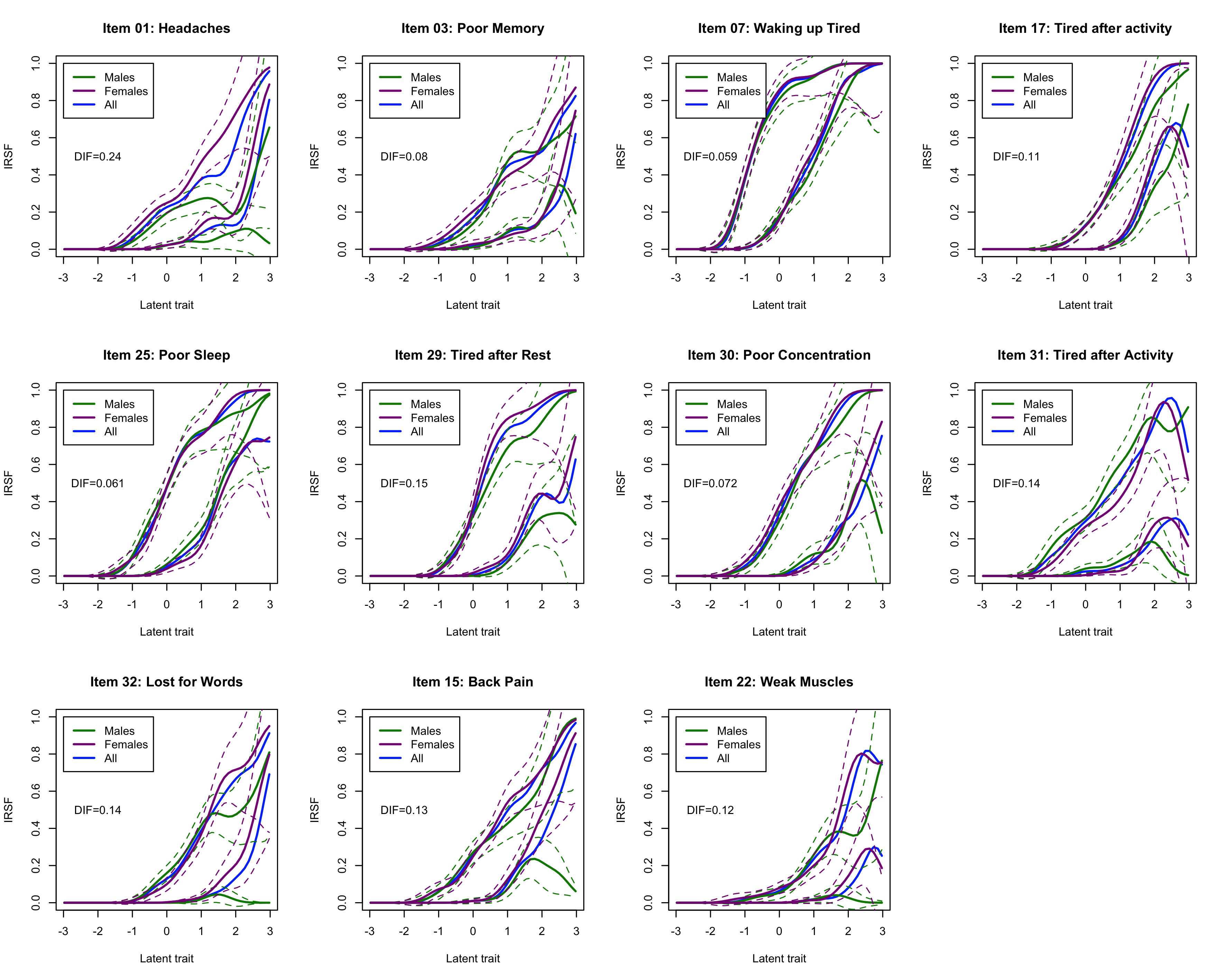

Supplement: Supplementary file 13 — Sex DIF for the 11 items of the chronic fatigue scale (TM wave). (PNG 1339 kb) [file 12888_2017_1420_MOESM13_ESM.png]

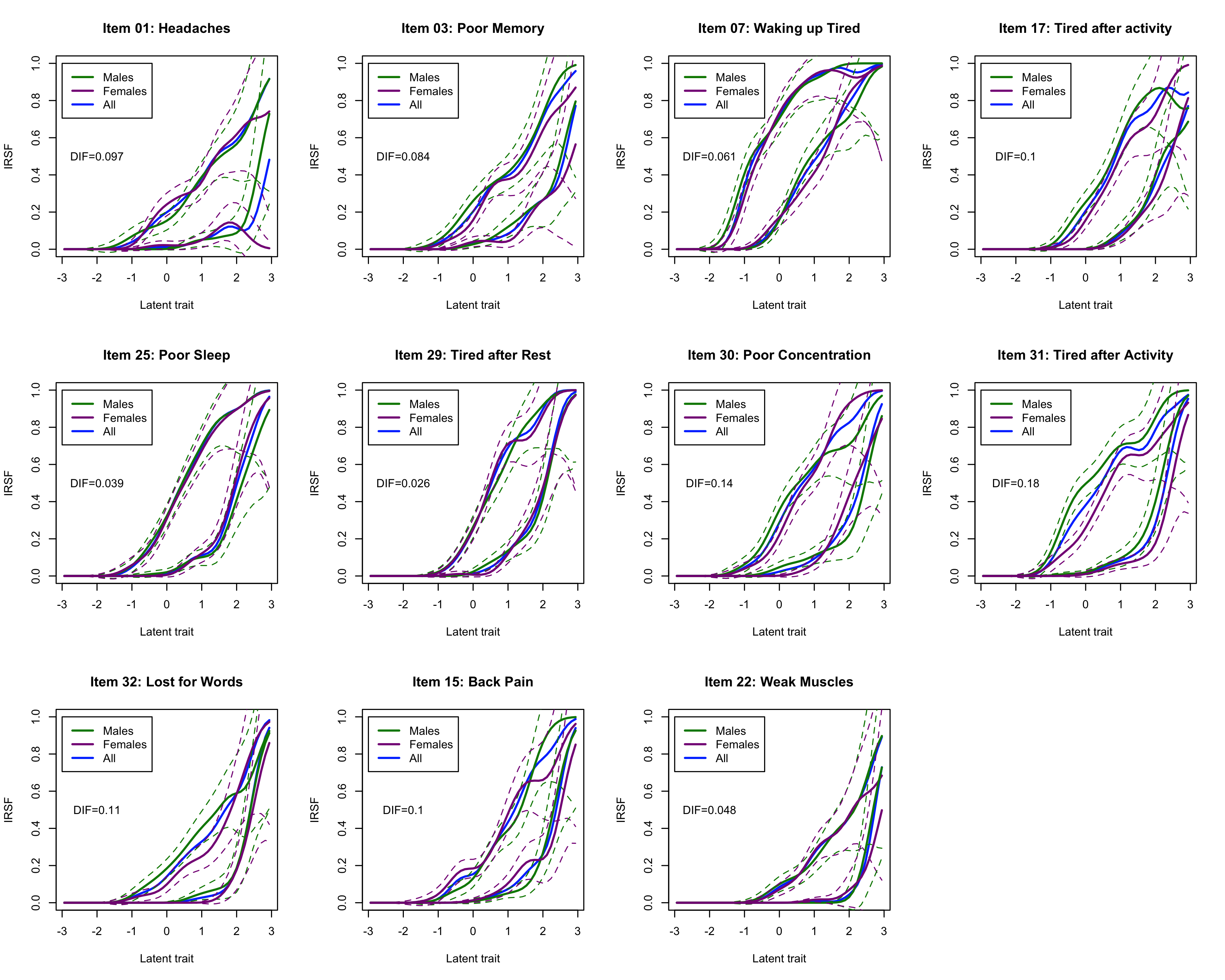

Supplement: Supplementary file 14 — Sex DIF for the 11 items of the chronic fatigue scale (TW2 wave). (PNG 1358 kb) [file 12888_2017_1420_MOESM14_ESM.png]

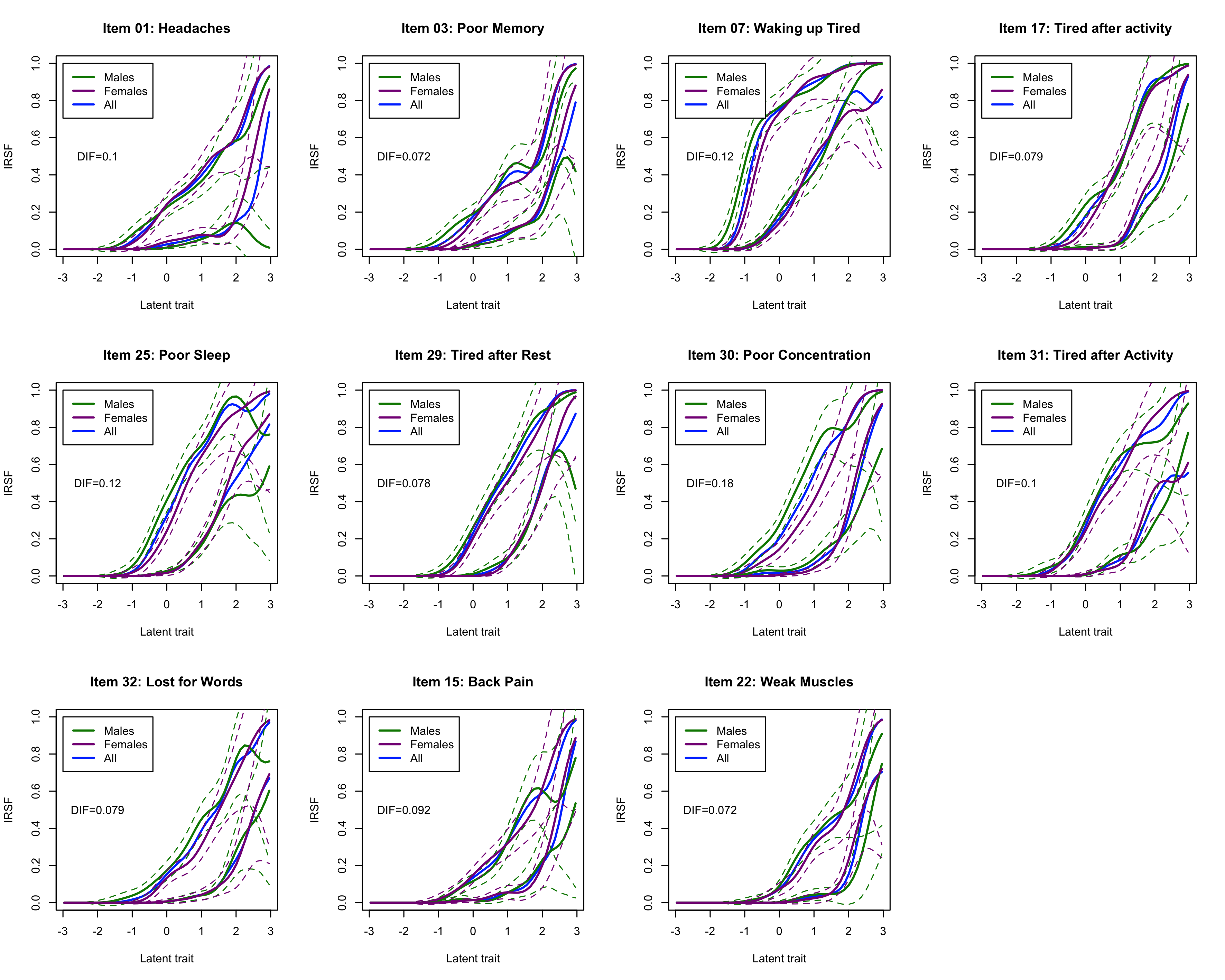

Supplement: Supplementary file 15 — Sex DIF for the 11 items of the chronic fatigue scale (TW1 wave). (PNG 1370 kb) [file 12888_2017_1420_MOESM15_ESM.png]

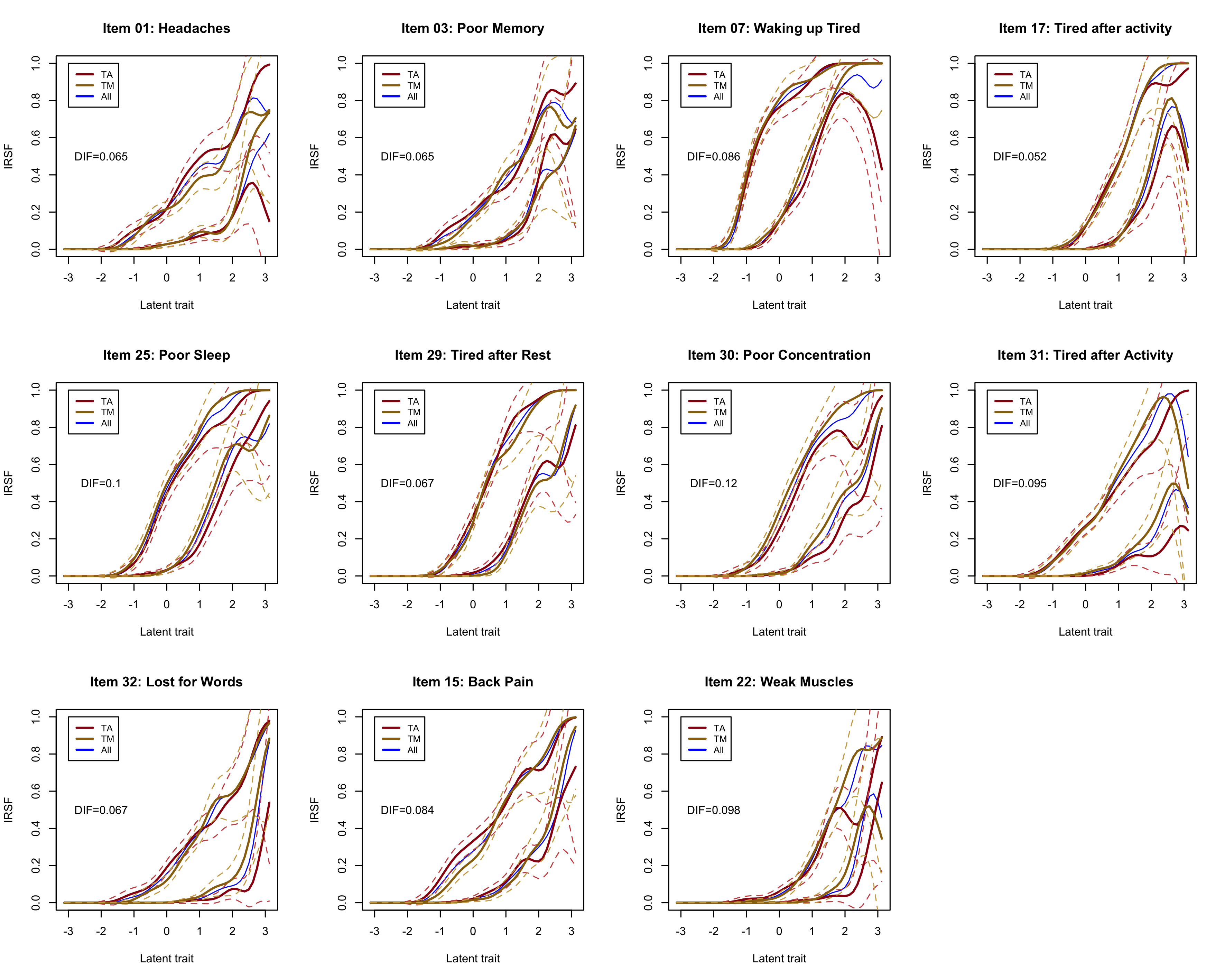

Supplement: Supplementary file 16 — DIF between studies TM and TA (chronic fatigue scale). (PNG 1342 kb) [file 12888_2017_1420_MOESM16_ESM.png]

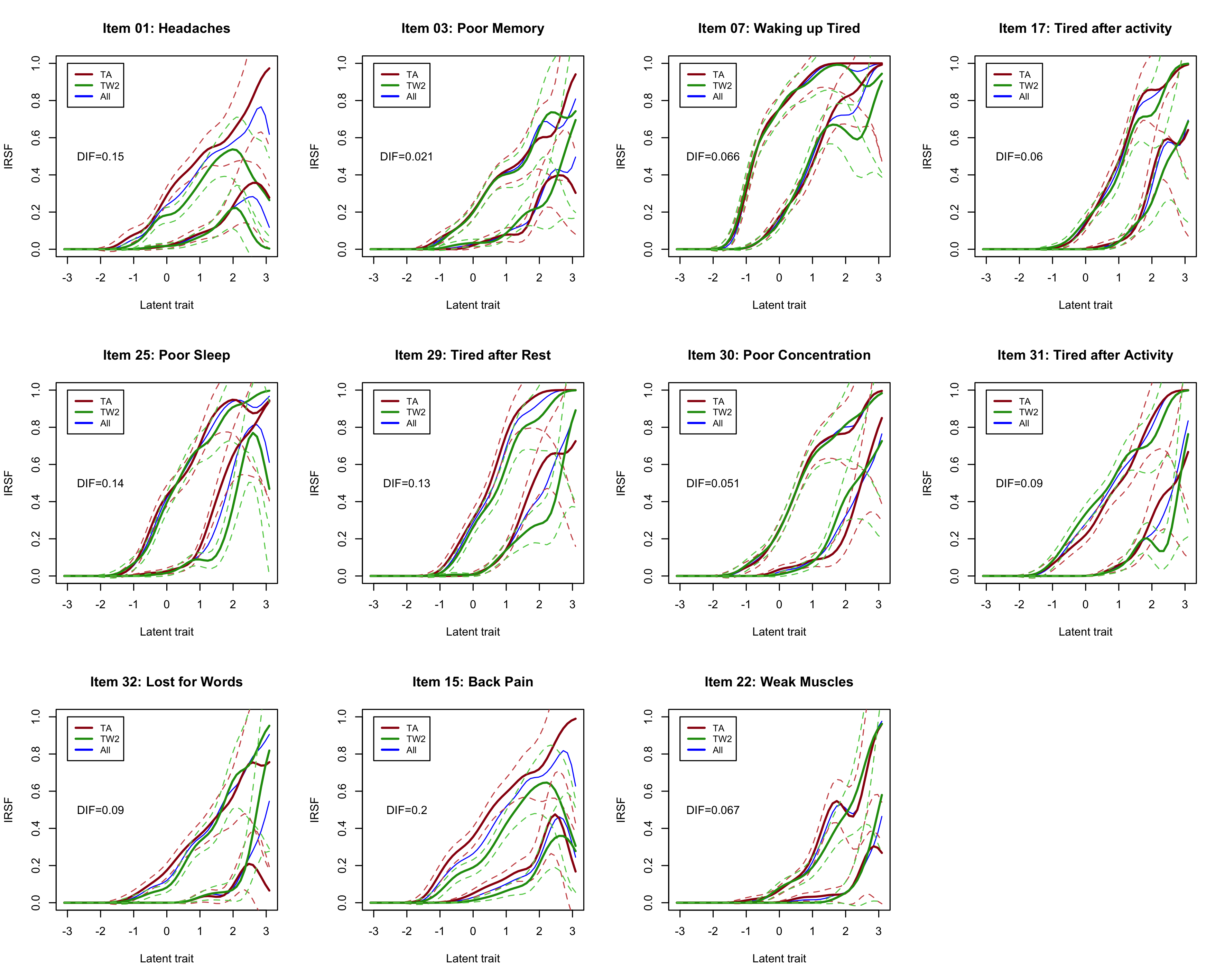

Supplement: Supplementary file 17 — DIF between studies TW2 and TA (chronic fatigue scale). (PNG 1330 kb) [file 12888_2017_1420_MOESM17_ESM.png]

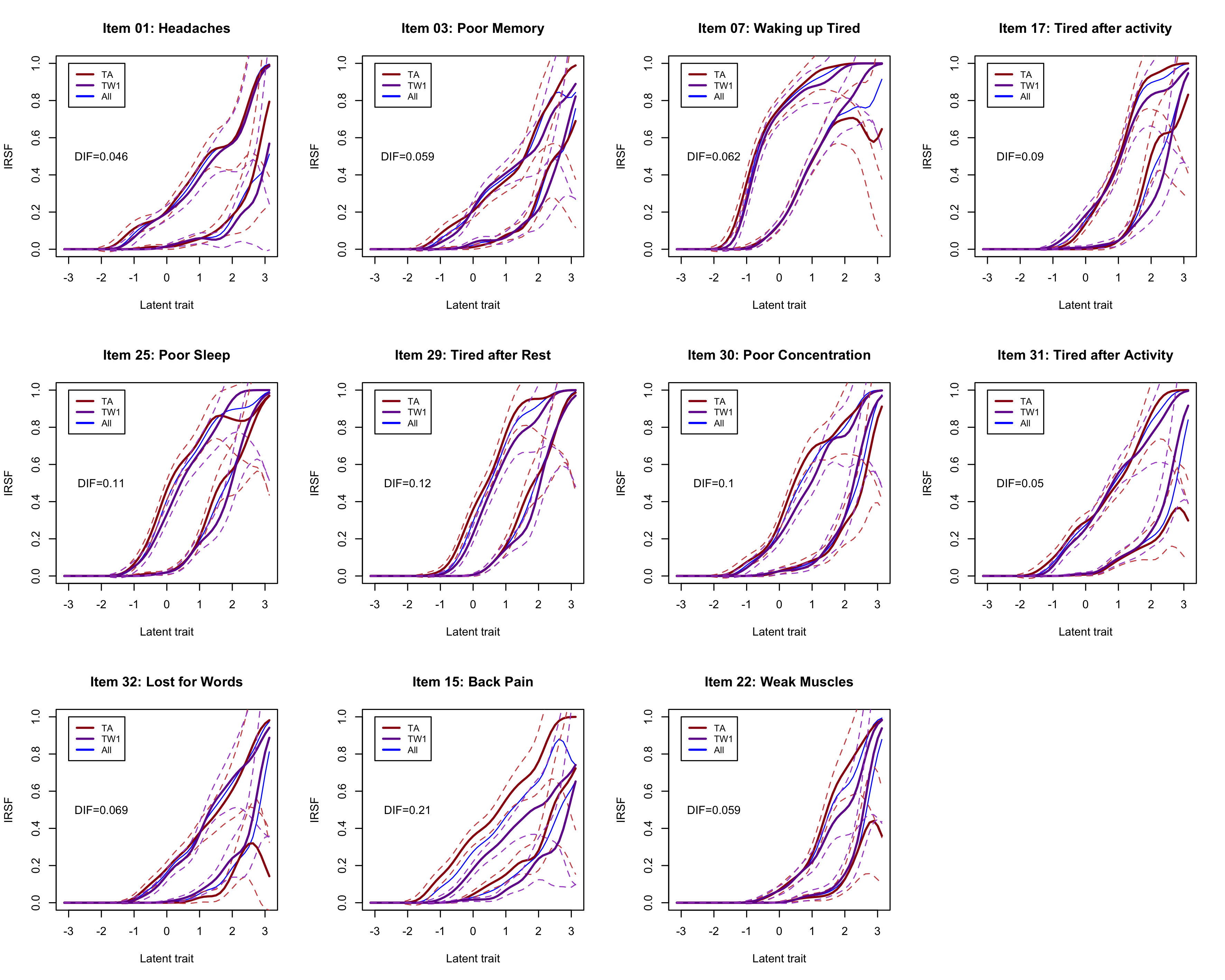

Supplement: Supplementary file 18 — DIF between studies TW1 and TA (chronic fatigue scale). (PNG 1350 kb) [file 12888_2017_1420_MOESM18_ESM.png]

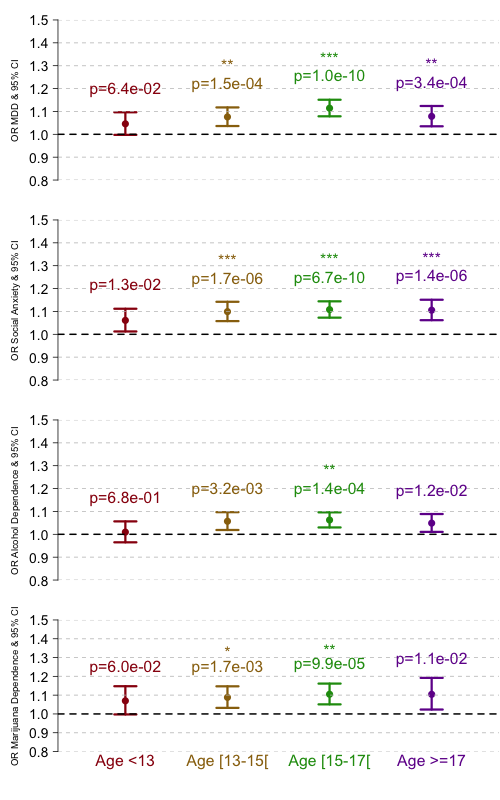

Supplement: Supplementary file 21 — Risk of MDD, social anxiety and substance dependence increases with anxiety-depression sum scores. (PNG 76 kb) [file 12888_2017_1420_MOESM21_ESM.png]

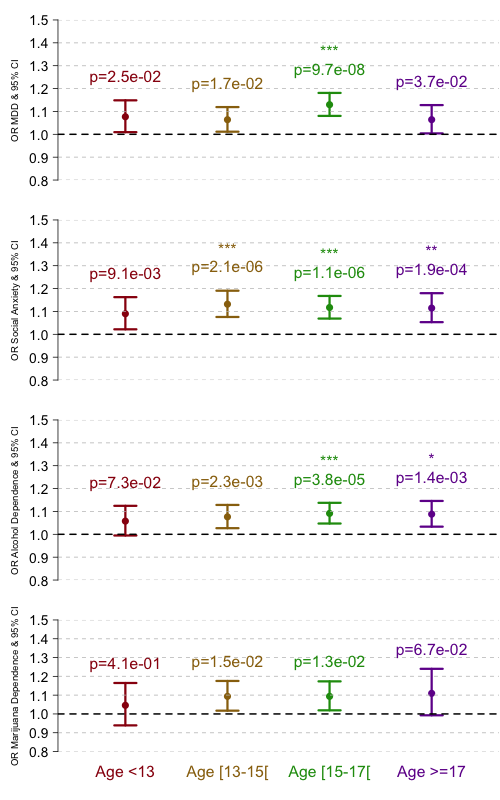

Supplement: Supplementary file 22 — Risk of MDD, social anxiety and substance dependence increases with chronic-fatigue sum scores. (PNG 77 kb) [file 12888_2017_1420_MOESM22_ESM.png]
