# Supplementary material for: Validation and psychometric properties of the Somatic and Psychological HEalth REport (SPHERE) in a young Australian-based population sample using non-parametric item response theory
Source: BMC Psychiatry. 2017 Aug 1;17:279. doi: 10.1186/s12888-017-1420-1 (PMC5540428; doi:10.1186/s12888-017-1420-1)
Supplement: Supplementary file 19 — SPHERE-21. (DOCX 22 kb) [file 12888_2017_1420_MOESM19_ESM.docx]

|  | | | | |
| --- | --- | --- | --- | --- |
|  | | | | |
| Please indicate if over the past few weeks you have been troubled by any of the following symptoms. Select the appropriate response to indicate **sometimes/never**, **often**, or **most of the time**. | | | | |
|  |  | | | |
|  |  | **Sometimes/Never** | **Often** | **Most of the time** |
| 1. | Feeling irritable or cranky? |  |  |  |
| 2. | Poor Memory? |  |  |  |
| 3. | Feeling nervous or tense? |  |  |  |
| 4. | Waking up tired? |  |  |  |
| 5. | Rapidly changing moods? |  |  |  |
| 6. | Feeling unhappy and depressed? |  |  |  |
| 7. | Back pain? |  |  |  |
| 8. | Prolonged tiredness after activity? |  |  |  |
| 9. | Feeling constantly under strain? |  |  |  |
| 10. | Weak muscles? |  |  |  |
| 11. | Feeling frustrated? |  |  |  |
| 12. | Poor sleep? |  |  |  |
| 13. | Getting annoyed easily? |  |  |  |
| 14. | Everything getting on top of you? |  |  |  |
| 15. | Dizziness? |  |  |  |
| 16. | Feeling tired after rest or relaxation? |  |  |  |
| 17. | Poor concentration? |  |  |  |
| 18. | Tired muscles after activity? |  |  |  |
| 19. | Feeling lost for words? |  |  |  |
| 20. | Losing confidence? |  |  |  |
| 21. | Being unable to overcome difficulties? |  |  |  |
