# Supplementary material for: Validation and psychometric properties of the Somatic and Psychological HEalth REport (SPHERE) in a young Australian-based population sample using non-parametric item response theory
Source: BMC Psychiatry. 2017 Aug 1;17:279. doi: 10.1186/s12888-017-1420-1 (PMC5540428; doi:10.1186/s12888-017-1420-1)
Supplement: Supplementary file 20 — Summary of genetic analyses: sampling homogeneity testing, ACE/ADE estimates and model fit comparison. (DOCX 197 kb) [file 12888_2017_1420_MOESM20_ESM.docx]

|  | score | Age | Sex | TW1 | TW2 | TM | Omnibus test  (20 df) | | H1c | H2c | H3c | H4c | rMZ | rDZ |
| --- | --- | --- | --- | --- | --- | --- | --- | --- | --- | --- | --- | --- | --- | --- |
|  |  | p-value | p-value | p-value | p-value | p-value | χ^2^ | p-value | p-value | p-value | p-value | p-value |  |  |
| **<13 years old** |  |  |  |  |  |  |  |  |  |  |  |  |  |  |
| Anxiety-depression | IRT | 0.86 | **4.8E-4** | 0.30 | 0.16 | NA | 29.3 | 0.082 | 0.21 | 0.33 | **9.4E-3** | **2.3E-14** | 0.43 | 0.22 |
|  | Sum score | 0.47 | **2.1E-4** | 0.74 | 0.32 | NA | 68.5 | **3.2E-7** | **1.8E-3** | 0.026 | **0.027** | **2.2E-20** | 0.46 | 0.28 |
| Chronic Fatigue | IRT | 0.58 | **1.2E-6** | 0.39 | 0.48 | NA | 26.8 | 0.14 | 0.16 | 0.19 | **0.032** | **2.4E-15** | 0.42 | 0.25 |
|  | Sum score | 0.55 | **5.4E-6** | 0.88 | 0.92 | NA | 47.8 | **4.5E-4** | **1.2E-3** | 0.02 | **0.027** | **2.6E-16** | 0.43 | 0.27 |
| **[13, 15[** |  |  |  |  |  |  |  |  |  |  |  |  |  |  |
| Anxiety-depression | IRT | 0.78 | 0.78 | 0.31 | 0.91 | NA | 13.5 | 0.85 | 0.69 | 0.81 | 0.78 | **1.3E-17** | 0.38 | 0.32 |
|  | Sum score | 0.32 | 0.96 | 0.99 | **0.049** | NA | 36.3 | **0.014** | 0.15 | 0.42 | 0.12 | **4.1E-12** | 0.34 | 0.24 |
| Chronic Fatigue | IRT | 0.22 | **0.019** | 0.11 | 0.52 | NA | 26.6 | 0.15 | 0.55 | 0.59 | 0.27 | **3.0E-21** | 0.53 | 0.29 |
|  | Sum score | 0.27 | 0.052 | 0.43 | 0.53 | NA | 36.6 | **0.013** | **6.7E-3** | 0.44 | **0.041** | **1.4E-18** | 0.48 | 0.25 |
| **[15, 17[** |  |  |  |  |  |  |  |  |  |  |  |  |  |  |
| Anxiety-depression | IRT | 0.33 | **5.4E-7** | 0.25 | 0.071 | 0.91 | 16.5 | 0.68 | 0.59 | 0.62 | 0.35 | **4.5E-10** | 0.28 | 0.20 |
|  | Sum score | 0.12 | **2.4E-10** | 0.77 | 0.32 | 0.21 | 53.9 | **5.9E-5** | 0.32 | 0.77 | 0.40 | **3.6E-7** | 0.24 | 0.15 |
| Chronic Fatigue | IRT | 0.08 | **0.017** | 0.12 | 0.16 | 0.19 | 13.6 | 0.85 | 0.25 | 0.91 | **0.045** | **1.10E-10** | 0.38 | 0.15 |
|  | Sum score | **0.04** | **0.038** | 0.34 | 0.41 | 0.059 | 18.0 | 0.59 | 0.63 | 0.89 | **0.016** | **4.6E-9** | 0.39 | 0.13 |
| **>=17** |  |  |  |  |  |  |  |  |  |  |  |  |  |  |
| Anxiety-depression | IRT | 0.06 | **5.1E-4** | 0.22 | NA | 0.53 | 19.7 | 0.48 | 0.95 | 0.81 | **0.022** | **4.6E-4** | 0.39 | 0.18 |
|  | Sum score | **0.049** | **1.7E-5** | 0.17 | NA | 0.15 | 48.8 | **3.3E-4** | 0.51 | 0.37 | 0.067 | 0.17 | 0.29 | 003 |
| Chronic Fatigue | IRT | **1.2E-3** | **2.0E-3** | 0.77 | NA | 0.11 | 27.5 | 0.12 | 0.58 | 0.97 | **0.042** | **4.4E-3** | 0.27 | 0.07 |
|  | Sum score | **2.0E-3** | **0.021** | 0.17 | NA | 0.52 | 36.1 | **0.015** | 0.18 | 0.44 | **0.01** | 0.1 | 0.26 | 0.01 |

Supplementary Table 1: Sampling homogeneity, covariate significance and twin pair correlations

P-values correspond to tests of likelihood ratio calculated in OpenMx. χ^2^ is the test statistic of the omnibus test (saturated model vs. reduced model with all means and variances equals across twin zygosity groups and siblings). rMZ and rDZ and the twin pair correlations for the MZ and DZ groups. H1c and H2c test equality of covariances that indicate sex specific heritability (scalar and non-scalar sex limitation), H3c is a test of familial aggregation and H4c a test of significance of the twin pair covariance. Notations correspond to those used in [1]. Nominally significant (p-value<0.05) tests are highlighted in bold.

Supplementary Table 2: Heritability of Anxiety-Depression IRT score across age groups

|  | **Model**† | **Parameter estimates** | | | **Model fit** | | | | | |
| --- | --- | --- | --- | --- | --- | --- | --- | --- | --- | --- |
|  |  | **A** | **C/D** | **E** | **df** | **Δdf** | **-2LL** | **Δ-2LL** | **AIC** | **P-value** |
| **<13** | ADE | 0.33 [0.058,0.49] | 0.081 [0.00,0.41] | 0.59 [0.5,0.68] | 7 |  | 5217.8 |  | 2467.8 | NA |
|  | **AE** | **0.41 [0.32,0.49]** |  | **0.59 [0.51,0.68]** | **6** | **1** | **5218** | **0.2** | **2466** | **0.65** |
|  | E |  |  |  | 5 | 2 | 5284.4 | 66.6 | 2530.4 | 3.50E-15 |
|  | ACE | 0.36 [0.15,0.49] | 0.035 [0.00,0.23] | 0.6 [0.51,0.71] | 7 |  | 5217.9 |  | 2467.9 |  |
| **[13-15[** | ACE | 0.19 [0.00,0.43] | 0.19 [0.00,0.35] | 0.62 [0.53,0.74] | 7 |  | 5193 |  | 2467 | NA |
|  | AE | 0.42 [0.33,0.5] |  | 0.58 [0.5,0.67] | 6 | 1 | 5196.9 | 3.9 | 2468.9 | 0.047 |
|  | **CE** |  | **0.31 [0.24,0.37]** | **0.69 [0.63,0.76]** | **6** | **1** | **5195.1** | **2.1** | **2467.1** | **0.15** |
|  | E |  |  |  | 5 | 2 | 5268.5 | 75.5 | 2538.5 | 4.10E-17 |
|  | ADE | 0.42 [0.28,0.5] | 0.00 [0.00,0.13] | 0.58 [0.5,0.67] | 7 |  | 5196.9 |  | 2470.9 |  |
| **[15-17[** | ACE | 0.13 [0.00,0.36] | 0.12 [0.00,0.27] | 0.74 [0.64,0.85] | 7 |  | 5732.7 |  | 2740.7 | NA |
|  | AE | 0.29 [0.2,0.38] |  | 0.71 [0.62,0.8] | 6 | 1 | 5734.2 | 1.5 | 2740.2 | 0.22 |
|  | **CE** |  | **0.21 [0.14,0.27]** | **0.79 [0.73,0.86]** | **6** | **1** | **5733.6** | **0.9** | **2739.6** | **0.34** |
|  | E |  |  |  | 5 | 2 | 5771 | 38.3 | 2775 | 5.00E-09 |
|  | ADE | 0.29 [0.16,0.38] | 0.00 [0.00,0.29] | 0.71 [0.62,0.8] | 7 |  | 5734.2 |  | 2742.2 |  |
| **17+** | ADE | 0.17 [0.00,0.49] | 0.22 [0.00,0.51] | 0.6 [0.46,0.77] | 8 |  | 3244.3 |  | 1526.3 | NA |
|  | **AE** | **0.37 [0.21,0.51]** |  | **0.63 [0.49,0.79]** | **7** | **1** | **3245.3** | **1** | **1525.3** | **0.32** |
|  | E |  |  |  | 6 | 2 | 3264.1 | 19.8 | 1542.1 | 5.10E-05 |
|  | ACE | 0.37 [0.09,0.51] | 0.00 [0.00,0.17] | 0.63 [0.49,0.79] | 8 |  | 3245.3 |  | 1527.3 |  |

Supplementary Table 3: Heritability of Anxiety-Depression sum score across age groups

|  | **Model**† | **Parameter estimates** | | | **Model fit** | | | | | |
| --- | --- | --- | --- | --- | --- | --- | --- | --- | --- | --- |
|  |  | **A** | **C/D** | **E** | **df** | **Δdf** | **-2LL** | **Δ-2LL** | **AIC** | **P-value** |
| **<13** | ACE | 0.30 [0.053,0.52] | 0.14 [0.00,0.32] | 0.56 [0.47,0.66] | 7 |  | 7914 |  | 5164.0 | NA |
|  | AE | **0.47 [0.38,0.55]** |  | **0.53 [0.45,0.62]** | **6** | **1** | **7916** | **2** | **5164.0** | **0.16** |
|  | CE |  | 0.34 [0.27,0.41] | 0.66 [0.59,0.73] | 6 | 1 | 7919.6 | 5.6 | 5167.6 | 0.018 |
|  | E |  |  |  | 5 | 2 | 8006.8 | 92.8 | 5252.8 | 7.20E-21 |
|  | ADE | 0.47 [0.38,0.55] | 0.00 [NA,0.27] | 0.53 [0.45,0.62] | 7 |  | 7916 |  | 5166.0 |  |
| **[13-15[** | ACE | 0.32 [0.025,0.48] | 0.053 [0.00,0.25] | 0.63 [0.52,0.76] | 7 |  | 7789.6 |  | 5063.6 | NA |
|  | AE | **0.39 [0.29,0.48]** |  | **0.61 [0.52,0.71]** | **6** | **1** | **7789.9** | **0.3** | **5061.9** | **0.58** |
|  | CE |  | 0.25 [0.18,0.32] | 0.75 [0.68,0.82] | 6 | 1 | 7794.1 | 4.5 | 5066.1 | 0.034 |
|  | E |  |  |  | 5 | 2 | 7841.7 | 52.1 | 5111.7 | 5.00E-12 |
|  | ADE | 0.39 [0.15,0.48] | 0.00 [0.00,0.25] | 0.61 [0.52,0.71] | 7 |  | 7789.9 |  | 5063.9 |  |
| **[15-17[** | ACE | 0.18 [0.00,0.34] | 0.052 [0.00,0.22] | 0.77 [0.66,0.89] | 7 |  | 8831.4 |  | 5839.4 | NA |
|  | AE | **0.25 [0.16,0.35]** |  | **0.75 [0.65,0.84]** | **6** | **1** | **8831.7** | **0.3** | **5837.7** | **0.6** |
|  | CE |  | 0.17 [0.1,0.23] | 0.83 [0.77,0.9] | 6 | 1 | 8833 | 1.6 | 5839.0 | 0.21 |
|  | E |  |  |  | 5 | 2 | 8857.4 | 26 | 5861.4 | 2.30E-06 |
|  | ADE | 0.25 [0.12,0.35] | 0.00 [0.00,0.24] | 0.75 [0.65,0.84] | 7 |  | 8831.7 |  | 5839.7 |  |
| **17+** | ADE | 0.053 [0.00,0.34] | 0.15 [0.00,0.36] | 0.79 [0.62,0.97] | 8 |  | 5097.9 |  | 3379.9 | NA |
|  | AE | 0.19 [0.022,0.36] |  | 0.81 [0.64,0.98] | 7 | 1 | 5098.6 | 0.7 | 3378.6 | 0.39 |
|  | **E** |  |  |  | **6** | **2** | **5103.5** | **5.6** | **3381.5** | **0.06** |
|  | ACE | 0.19 [0.022,0.36] | 0.00 [0.00,0.14] | 0.81 [0.64,0.98] | 8 |  | 5098.6 |  | 3380.6 |  |

Supplementary Table 4: Heritability of Chronic-Fatigue IRT score across age groups

|  | **Model**† | **Parameter estimates** | | | **Model fit** | | | | | |
| --- | --- | --- | --- | --- | --- | --- | --- | --- | --- | --- |
|  |  | **A** | **C/D** | **E** | **df** | **Δdf** | **-2LL** | **Δ-2LL** | **AIC** | **P-value** |
| **<13** | ACE | 0.37 [0.11,0.51] | 0.046 [0.00,0.23] | 0.59 [0.49,0.69] | 7 |  | 5111.3 |  | 2361.3 | NA |
|  | AE | **0.42 [0.33,0.51]** |  | **0.58 [0.49,0.67]** | **6** | **1** | **5111.5** | **0.2** | **2359.5** | **0.64** |
|  | CE |  | 0.29 [0.22,0.36] | 0.71 [0.64,0.78] | 6 | 1 | 5118.9 | 7.6 | 2366.9 | 0.0058 |
|  | E |  |  |  | 5 | 2 | 5181.7 | 70.4 | 2427.7 | 5.10E-16 |
|  | ADE | 0.42 [0.26,0.51] | 0.00 [0.00,0.24] | 0.58 [0.49,0.67] | 7 |  | 5111.5 |  | 2361.5 |  |
| **[13-15[** | ACE | 0.49 [0.26,0.59] | 0.021 [0.00,0.19] | 0.49 [0.41,0.59] | 7 |  | 4900.5 |  | 2174.5 | NA |
|  | AE | **0.51 [0.43,0.59]** |  | **0.49 [0.41,0.57]** | **6** | **1** | **4900.6** | **0.1** | **2172.6** | **0.81** |
|  | CE |  | 0.33 [0.27,0.4] | 0.67 [0.6,0.73] | 6 | 1 | 4917.2 | 16.7 | 2189.2 | 4.50E-05 |
|  | E |  |  |  | 5 | 2 | 5007.7 | 107 | 2277.7 | 5.30E-24 |
|  | ADE | 0.51 [0.43,0.59] | 0.00 [0.00,0.1] | 0.49 [0.41,0.57] | 7 |  | 4900.6 |  | 2174.6 |  |
| **[15-17[** | ACE | 0.35 [0.15,0.44] | 0.00 [0.00,0.13] | 0.65 [0.56,0.75] | 7 |  | 5322.7 |  | 2330.7 | NA |
|  | AE | **0.35 [0.25,0.44]** |  | **0.65 [0.56,0.75]** | **6** | **1** | **5322.7** | **0** | **2328.7** | **1** |
|  | CE |  | 0.22 [0.15,0.28] | 0.78 [0.72,0.85] | 6 | 1 | 5332.4 | 9.7 | 2338.4 | 0.0018 |
|  | E |  |  |  | 5 | 2 | 5372.4 | 49.7 | 2376.4 | 1.60E-11 |
|  | ADE | 0.35 [0.11,0.44] | 0.00 [0.00,0.25] | 0.65 [0.56,0.75] | 7 |  | 5322.7 |  | 2330.7 |  |
| **17+** | ADE | 0.00 [0.00,0.36] | 0.32 [0.00,0.46] | 0.68 [0.54,0.85] | 8 |  | 3031.3 |  | 1313.3 | NA |
|  | AE | **0.27 [0.11,0.41]** |  | **0.73 [0.59,0.89]** | **7** | **1** | **3033.6** | **2.3** | **1313.6** | **0.13** |
|  | E |  |  |  | 6 | 2 | 3044.2 | 12.9 | 1322.2 | 0.0015 |
|  | ACE | 0.27 [0.00,0.41] | 0.00 [0.00,0.23] | 0.73 [0.59,0.89] | 8 |  | 3033.6 |  | 1315.6 |  |

Supplementary Table 5: Heritability of Chronic-Fatigue sum score across age groups

|  | **Model**† | **Parameter estimates** | | | **Model fit** | | | | | |
| --- | --- | --- | --- | --- | --- | --- | --- | --- | --- | --- |
|  |  | **A** | **C/D** | **E** | **df** | **Δdf** | **-2LL** | **Δ-2LL** | **AIC** | **P-value** |
| **<13** | ACE | 0.40 [0.15,0.53] | 0.039 [0.00,0.23] | 0.56 [0.47,0.66] | 7 |  | 7103.3 |  | 4353.3 | NA |
|  | AE | **0.45 [0.36,0.53]** |  | **0.55 [0.47,0.64]** | **6** | **1** | **7103.4** | **0.1** | **4351.4** | **0.69** |
|  | CE |  | 0.31 [0.24,0.37] | 0.69 [0.63,0.76] | 6 | 1 | 7112.3 | 9 | 4360.3 | 0.0026 |
|  | E |  |  |  | 5 | 2 | 7179.9 | 76.6 | 4425.9 | 2.40E-17 |
|  | ADE | 0.45 [0.29,0.53] | 0.00 [0.00,0.23] | 0.55 [0.47,0.64] | 7 |  | 7103.4 |  | 4353.4 |  |
| **[13-15[** | ACE | 0.46 [0.21,0.57] | 0.021 [0.00,0.2] | 0.52 [0.43,0.63] | 7 |  | 7106.5 |  | 4380.5 | NA |
|  | AE | **0.49 [0.4,0.57]** |  | **0.51 [0.43,0.6]** | **6** | **1** | **7106.6** | **0.1** | **4378.6** | **0.81** |
|  | CE |  | 0.31 [0.24,0.37] | 0.69 [0.63,0.76] | 6 | 1 | 7118.9 | 12.4 | 4390.9 | 0.00044 |
|  | E |  |  |  | 5 | 2 | 7196.3 | 89.8 | 4466.3 | 3.20E-20 |
|  | ADE | 0.49 [0.29,0.57] | 0.00 [0.00,0.2] | 0.51 [0.43,0.6] | 7 |  | 7106.6 |  | 4380.6 |  |
| **[15-17[** | ADE | 0.33 [0.076,0.44] | 0.023 [0.00,0.28] | 0.65 [0.56,0.75] | 7 |  | 7963.2 |  | 4971.2 | NA |
|  | AE | **0.35 [0.25,0.44]** |  | **0.65 [0.56,0.75]** | **6** | **1** | **7963.2** | **0** | **4969.2** | **0.87** |
|  | E |  |  |  | 5 | 2 | 8009 | 45.8 | 5013 | 1.10E-10 |
|  | ACE | 0.35 [0.19,0.44] | 0.00 [0.00,0.099] | 0.65 [0.56,0.75] | 7 |  | 7963.2 |  | 4971.2 |  |
| **17+** | ADE | 0.00 [0.00,0.24] | 0.24 [0.048,0.39] | 0.76 [0.61,0.93] | 8 |  | 4526.4 |  | 2808.4 | NA |
|  | AE | **0.18 [0.015,0.35]** |  | **0.82 [0.65,0.99]** | **7** | **1** | **4529.9** | **3.5** | **2809.9** | **0.062** |
|  | E |  |  |  | 6 | 2 | 4534.5 | 8.1 | 2812.5 | 0.018 |
|  | ACE | 0.18 [0.00,0.35] | 0.00 [0.00,0.17] | 0.82 [0.65,0.99] | 8 |  | 4529.9 |  | 2811.9 |  |

Supplementary Table 6: Phenotypic, genetic and environmental correlations between Anxiety-depression and Chronic-fatigue IRT scores

|  | Phenotypic correlation | | | | Genetic correlation | | | Environmental correlation | | | |
| --- | --- | --- | --- | --- | --- | --- | --- | --- | --- | --- | --- |
|  | r [95% CI] | p-value  (H0: r=0) | p-value  (H0: r=1) | r [95% CI] | | p-value (H0: r=0) | p-value  (H0: r=1) | r [95% CI] | p-value (H0: r=0) | p-value (H0: r=1) |  |
| <13 | 0.62 [0.58,0.65] | **2.8E-128** | **2.1E-20** | 0.88 [0.77,0.98] | | **8.2E-18** | 0.17 | 0.44 [0.36,0.52] | **1.7E-23** | **2.3E-12** |  |
| [13,15[ | 0.67 [0.64,0.70] | **6.1E-149** | **1.5E-21** | 0.85 [0.77,0.92] | | **1.4E-20** | 0.15 | 0.52 [0.43,0.59] | **2.4E-28** | **4.1E-14** |  |
| [15,17[ | 0.68 [0.65,0.70] | **9.9E-176** | **1.4E-19** | 0.88 [0.77,0.97] | | **8.1E-11** | **7.0E-4** | 0.58 [0.51,0.64] | **2.3E-52** | **2.6E-8** |  |
| >=17 | 0.63 [0.57,0.68] | **1.8E-65** | **8.6E-17** | 1.00 [0.88,1.00] | | **5.2E-7** | 0.017 | 0.43 [0.31,0.54] | **8.0E-11** | **1.5E-6** |  |

The p-values correspond to a likelihood ratio test with 1 df for tests on genetic and environmental correlations, 2 df for phenotypic correlations. The null hypothesis is indicated in brackets. Significant tests after Bonferroni correction (16 tests, significance threshold of 0.003) appear in bold.

Supplementary Table 7: Phenotypic, genetic and environmental correlations between Anxiety-depression and Chronic-fatigue sum scores

|  | Phenotypic correlation | | | | Genetic correlation | | | Environmental correlation | | | |
| --- | --- | --- | --- | --- | --- | --- | --- | --- | --- | --- | --- |
|  | r [95% CI] | p-value  (H0: r=0) | p-value  (H0: r=1) | r [95% CI] | | p-value (H0: r=0) | p-value  (H0: r=1) | r [95% CI] | p-value (H0: r=0) | p-value (H0: r=1) |  |
| <13 | 0.73 [0.71,0.76] | **5.1E-207** | **<1.0E-300** | 0.87 [0.79,0.93] | | **1.9E-19** | **7.7E-8** | 0.63 [0.56,0.69] | 1.9E-53 | **7.8E-164** |  |
| [13,15[ | 0.70 [0.67,0.73] | **9.5E-174** | **3.2E-31** | 0.83 [0.74,0.90] | | **3.9E-14** | **2.6E-6** | 0.61 [0.54,0.68] | 2.0E-41 | **1.1E-4** |  |
| [15,17[ | 0.69 [0.66,0.72] | **2.1E-191** | **2.1E-41** | 0.76 [0.61,0.87] | | **1.0E-6** | 0.020 | 0.67 [0.61,0.72] | 4.2E-66 | **4.1E-13** |  |
| >=17 | 0.66 [0.62,0.70] | **2.6E-75** | **2.2E-11** | 1.00 [0.92,1.00] | | **4.0E-4** | 0.042 | 0.53 [0.43,0.62] | 1.6E-18 | 0.013 |  |

The p-values correspond to a likelihood ratio test with 1 df for tests on genetic and environmental correlations, 2 df for phenotypic correlations. The null hypothesis is indicated in brackets. Significant tests after Bonferroni correction (16 tests, significance threshold of 0.003) appear in bold.

Supplementary Table 8: Phenotypic, genetic and environmental correlations between Anxiety-depression and Chronic-fatigue IRT scores after removing items in common from the anxiety-depression scale

|  | Phenotypic correlation | | | | Genetic correlation | | | Environmental correlation | | | |
| --- | --- | --- | --- | --- | --- | --- | --- | --- | --- | --- | --- |
|  | r [95% CI] | p-value  (H0: r=0) | p-value  (H0: r=1) | r [95% CI] | | p-value (H0: r=0) | p-value  (H0: r=1) | r [95% CI] | p-value (H0: r=0) | p-value (H0: r=1) |  |
| <13 | 0.55 [0.51,0.58] | **8.9E-94** | **2.8E-25** | 0.83 [0.71,0.95] | | **1.4E-6** | 0.057 | 0.36 [0.27,0.45] | **1.1E-13** | **9.4E-17** |  |
| [13,15[ | 0.57 [0.53,0.61] | **5.2E-100** | **5.7E-28** | 0.87 [0.77,0.99] | | **1.5E-19** | 0.40 | 0.36 [0.26,0.45] | **2.2E-13** | **2.0E-22** |  |
| [15,17[ | 0.60 [0.56,0.63] | **3.8E-127** | **8.1E-25** | 0.91 [0.75,1.00] | | **7.7E-10** | 4.7E-3 | 0.48 [0.40,0.55] | **9.2E-34** | **2.6E-16** |  |
| >=17 | 0.56 [0.50,0.62] | **8.1E-49** | **3.8E-22** | 0.97 [0.75,1.00] | | **1.4E-6** | 4.7E-3 | 0.35 [0.20,0.49] | **2.0E-6** | **1.6E-7** |  |

The p-values correspond to a likelihood ratio test with 1 df for tests on genetic and environmental correlations, 2 df for phenotypic correlations. The null hypothesis is indicated in brackets. Significant tests after Bonferroni correction (16 tests, significance threshold of 0.003) appear in bold.

1. Evans DM, Frazer IH, Martin NG: **Genetic and environmental causes of variation in basal levels of blood cells**. *Twin Res* 1999, **2**(4):250-257.
