# Supplementary material for: Validation and psychometric properties of the Somatic and Psychological HEalth REport (SPHERE) in a young Australian-based population sample using non-parametric item response theory
Source: BMC Psychiatry. 2017 Aug 1;17:279. doi: 10.1186/s12888-017-1420-1 (PMC5540428; doi:10.1186/s12888-017-1420-1)
Supplement: Supplementary file 23 — Glossary. (DOCX 92.5 kb) [file 12888_2017_1420_MOESM23_ESM.docx]

Glossary

**Conditional Independence:** IRT hypothesis that states that the answer to one item is independent to the other items’ answers, conditionally on the latent trait.  **Differential Item Functioning (DIF):** see Item bias

**Environmental correlation**: proportion of the variance shared between two traits that can be explained by common environmental sources.

**Fast Fourier transform:** a type of Fourier analysis used here to estimate the non-parametric IRSF. The basis of Fourier analysis is to convert a signal (or data) from its original domain to a representation in the frequency domain and vice versa. Some mathematical operations (such as integration) are much simpler in the frequency domain, and using Fourier transform can be seen as a mathematical shortcut that speeds up the estimation of parameters/likelihood.

**Genetic correlation:** proportion of the variance shared between two traits that can be explained by common genetic sources.

**Genetic relatedness / kinship coefficient:** measure of genetic proximity or consanguinity between two individuals. For example the kinship coefficient is 1 between MZ twins who are genetic clones, 0.5 between DZ twins who share on average 50% of their DNA information.

**Item bias:** an item characteristic in IRT. Item bias quantifies if the item might measure different abilities for members of subgroups (e.g. sex or age groups). Items with large DIF correspond to items understood or answered differently across age or sex groups, whose exclusion should be discussed.

**Kernel regression**: a non-parametric statistical technique whose objective is to model a relationship between 2 random variables (here the relationships between items response and the latent trait) without making hypotheses on the shape of the relationship.

**Monotonicity (of the IRSF)**: IRT hypothesis that states that all the IRSF should be monotonous (growing) functions of the latent trait.

**Phenotypic correlation**: correlation between two observed measurements. The phenotypic correlation is the weighted mean of the genetic and environmental correlations.
**Sex limitation:** in twin models, presence of sex differences in heritability that arise from specific sources of variance in each sex group or from differences in amount of variance explained (by the same sources of variance).

**Stochastic ordering (on the latent trait) by the sum score**: the order of the examinees on the sum score gives a stochastically correct ordering of the examinees on the latent variable. This allows making inference on the latent trait based on the ordering provided by the sum score.
**Unidimensionality**: IRT hypothesis that states that a single dimension underlies the participants’ responses to the questionnaire.
